# Upregulation of ERK-EGR1-heparanase axis by HDAC inhibitors provides targets for rational therapeutic intervention in synovial sarcoma

**DOI:** 10.1186/s13046-021-02150-y

**Published:** 2021-12-02

**Authors:** Cinzia Lanzi, Enrica Favini, Laura Dal Bo, Monica Tortoreto, Noemi Arrighetti, Nadia Zaffaroni, Giuliana Cassinelli

**Affiliations:** Department of Applied Research and Technological Development, Molecular Pharmacology Unit, Fondazione IRCCS Istituto Nazionale dei Tumori, Via Amadeo 42, 20133 Milan, Italy

**Keywords:** EGR1, ERK, Heparanase inhibitor, Histone deacetylase inhibitor, FK228, SAHA, SST0001, Synovial sarcoma

## Abstract

**Background:**

Synovial sarcoma (SS) is an aggressive soft tissue tumor with limited therapeutic options in advanced stage. *SS18-SSX* fusion oncogenes, which are the hallmarks of SS, cause epigenetic rewiring involving histone deacetylases (HDACs). Promising preclinical studies supporting HDAC targeting for SS treatment were not reflected in clinical trials with HDAC inhibitor (HDACi) monotherapies. We investigated pathways implicated in SS cell response to HDACi to identify vulnerabilities exploitable in combination treatments and improve the therapeutic efficacy of HDACi-based regimens.

**Methods:**

Antiproliferative and proapoptotic effects of the HDACi SAHA and FK228 were examined in SS cell lines in parallel with biochemical and molecular analyses to bring out cytoprotective pathways. Treatments combining HDACi with drugs targeting HDACi-activated prosurvival pathways were tested in functional assays in vitro and in a SS orthotopic xenograft model*.* Molecular mechanisms underlying synergisms were investigated in SS cells through pharmacological and gene silencing approaches and validated by qRT-PCR and Western blotting.

**Results:**

SS cell response to HDACi was consistently characterized by activation of a cytoprotective and auto-sustaining axis involving ERKs, EGR1, and the β-endoglycosidase heparanase, a well recognized pleiotropic player in tumorigenesis and disease progression. HDAC inhibition was shown to upregulate heparanase by inducing expression of the positive regulator EGR1 and by hampering negative regulation by p53 through its acetylation. Interception of HDACi-induced ERK-EGR1-heparanase pathway by cell co-treatment with a MEK inhibitor (trametinib) or a heparanase inhibitor (SST0001/roneparstat) enhanced antiproliferative and pro-apoptotic effects. HDAC and heparanase inhibitors had opposite effects on histone acetylation and nuclear heparanase levels. The combination of SAHA with SST0001 prevented the upregulation of ERK-EGR1-heparanase induced by the HDACi and promoted caspase-dependent cell death. In vivo*,* the combined treatment with SAHA and SST0001 potentiated the antitumor efficacy against the CME-1 orthotopic SS model as compared to single agent administration.

**Conclusions:**

The present study provides preclinical rationale and mechanistic insights into drug combinatory strategies based on the use of ERK pathway and heparanase inhibitors to improve the efficacy of HDACi-based antitumor therapies in SS. The involvement of classes of agents already clinically available, or under clinical evaluation, indicates the transferability potential of the proposed approaches.

**Supplementary Information:**

The online version contains supplementary material available at 10.1186/s13046-021-02150-y.

## Background

Synovial sarcoma (SS) is a rare aggressive malignancy mainly occurring in adolescents and young adults. It is characterized by the pathognomonic reciprocal t(X;18)(p11.2;q11.2) translocation leading to the fusion of the *SS18* gene (HUGO Gene Nomenclature Committee, HGNC, ID 11340) with the *SSX1*, *SSX2* (HGNC IDs 11335, 11336) and, rarely, *SSX4* (HGNC ID 11338) genes. SS18-SSX fusion proteins exert an oncogenic activity through complex not yet fully elucidated mechanisms. Although devoid of DNA binding domains, the fusion partners cause aberrant activation or repression of gene transcription through an epigenetic rewiring. In fact, by interacting with components of the chromatin regulatory complex SWItch/Sucrose Non-Fermentable (SWI/SNF) and the histone modifiers Polycomb Repressive Complexes (PRC1 and PRC2), SS18-SSXs can alter their activities [1–3].

Despite multimodal treatments including surgery, radiotherapy and chemotherapy, SS remains a deadly disease with a 10-year survival rate of about 50% [4–6]. SS18-SSX oncoproteins are considered pharmacologically undruggable [7]. Mechanistic studies have identified several molecules/pathways deregulated by the chimeric protein activities as potential alternative therapeutic targets. Among these, receptor tyrosine kinases (e.g. PDGFR, IGF1R/IR), components of chromatin remodeling complexes (e.g. EZH2, BRD9, KDM2B, HDACs) and cell cycle regulators (e.g. CDKs) have been the objects of intense investigation [2, 3, 8–10]. Propensity toward angiogenesis and aberrant activation of PDGFR pathway in several soft tissue sarcomas [4, 7] has provided the rationale for the use of pazopanib which is currently approved for second-line treatment in advanced setting. Nonetheless, the impact of these targeted approaches for improving SS outcome is still limited [3–6].

Human histone deacetylases (HDACs) are a group of 18 enzymes, divided into four classes (I-IV) catalyzing the removal of acetyl groups from the lysine residues of both histone and non-histone proteins. Counteracting the action of histone acetyltransferases (HATs) that catalyze the reaction of lysine acetylation, HDACs participate in regulating chromatin structure, gene expression and a variety of cellular processes [8, 11]. HDACs have been reported to activate or repress the transcription of about 10% of total genes, including tumor suppressors and oncogenes [11, 12] thus contributing to govern a wide array of biological processes implicated in cancer initiation and progression [11].

The description of HDACs highly expressed in SS specimens and directly interacting with the SS18-SSX oncoproteins [9, 13–17] has paved the way to the evaluation of HDAC inhibitors (HDACi) in this malignancy. By virtue of the capability to target core mechanisms participating in SS cell transformation and the proapoptotic effect observed in some SS cell lines, HDACi have emerged as valuable therapeutics in the preclinical setting [10, 16–23]. Some of these agents have been approved for treatment of hematological malignancies such as cutaneous or refractory peripheral T cell lymphoma (i.e. vorinostat, romidepsin, belinostat) and multiple myeloma (panobinostat), and many other drugs of this class are under clinical evaluation [12, 24]. HDACi provided modest results in soft tissue sarcomas including SS [25, 26] and combinations with cytotoxic drugs are currently under clinical evaluation (www.clinicaltrial.gov). Despite mechanistic studies support the use of HDACi to treat SS, mechanisms of drug resistance remain largely unknown pointing out the need for identifying rationale-based combinations to improve antitumor efficacy of these agents.

Several lines of evidence have implicated the endo-β-D-glycosidase heparanase encoded by the *HPSE* gene (HGNC ID 5164) and its substrates, i.e. the heparan sulfate (HS) chains of HS proteoglycans (HSPGs), in critical processes of the pathobiology of several tumor types including sarcomas (e.g. growth, angiogenesis, inflammation, metastasis, drug resistance) [27–32]. A deregulated heparanase/HSPG system profoundly impacts on tumor aggressiveness by acting both in the tumor microenvironment and inside the tumor cells. Emerging evidence indicates that nuclear heparanase and HSPGs also play a role in regulating histone acetylation and gene expression [33–35]. A potential relationship between deregulated heparanase/HSPG axis and oncogenic players in the different sarcoma subtypes remains to be elucidated. Heparanase is expressed in SS cell lines and tumor specimens [36, 37] and preclinical studies using HS mimetics and small molecule heparanase inhibitors have indicated heparanase and HSPGs as druggable targets in different types of sarcoma models including SS [29, 37–40].

In this study, we explored pathways activated in the SS cell response to HDACi to investigate new combination treatments enhancing the drug proapoptotic effects. Our findings reveal the ERK-EGR1-heparanase axis as an auto-sustaining compensatory pathway activated by HDACi, and point to MEK and heparanase/HSPGs as targets for drug combinations with improved antitumor efficacy.

## Materials and methods

### Cell lines and culture conditions

The human SS cell lines SYO-1 [41] and MoJo [42], provided by K.B. Jones (University of Utah, Salt Lake City, UT), were cultured in DMEM medium (Lonza, Verviers, Belgium) supplemented with non-essential amino acids and 10% or 20% fetal bovine serum (FBS), respectively. CME-1 cells [43], provided by M. Pierotti (Fondazione Istituto FIRC Oncologia Molecolare, Milan, Italy), were maintained in RPMI medium (Lonza) and 10% FBS. Yamato-SS and Aska-SS cell lines, originally established by Naka et al. [44] and provided by Y.M.H. Versleijen-Jonkers (Radboud University Medical Center, Nijmegen, The Netherlands), were cultured in DMEM medium with 10% (Yamato-SS) or 20% (Aska-SS) FBS. The human SS cell lines 1273/99, donated by O. Larsson (Karolinska Institute, Stockholm, Sweden), were cultured in Ham’s F12 (Lonza) with 20% FBS [45]. The expression of the pathognomonic *SS18-SSX* fusion products in SS cell lines was confirmed and periodically controlled by RT-PCR (Supplementary Fig. S1a) and Western blot analysis as described [37]. Further details about the mutational status of SS cell lines are reported in [46–50].

### Drugs

The following commercially available reagents were used: the class I, II and IV HDACi suberanilohydroxamic acid (SAHA), the class I HDAC/PI3K inhibitor bicyclic depsipeptide (FK228), the glycosylation inhibitor tunicamycin, the MEK1/2 inhibitor trametinib, the small molecule heparanase inhibitor OGT2115, the MDM2-p53 binding inhibitor nutlin-3. The suppliers of these reagents are reported in Supplementary Table S1. The hydroxamate-based HDACi ST3595 was provided by Sigma-Tau Industrie Farmaceutiche Riunite S.p.A. (Pomezia, IT) [51]. For in vitro studies, these drugs were dissolved in DMSO and further diluted in cell culture medium (0.1–0.5% DMSO final concentration). The HS mimetic/heparanase inhibitor SST0001 (roneparstat, ^100^NA-ROH) [52], was provided by Leadiant Biosciences S.p.a., (Rome, IT); SST0762NA1, a biotinylated structural analog of SST0001, provided by G. Ronzoni Institute for Chemical and Biochemical Research (Milan, IT), was prepared by conjugation on NH_2_ group of glucosamine residues with biotin N-hydroxysuccinimide ester, as previously reported for compound B1 [40]. SST0762NA1 has Mw of 7800 Da and about 2 biotin moieties for heparin chain. For in vitro studies, SST0001 and SST0762NA1 were dissolved in sterile water.

### Cellular studies

Cells were treated with drugs after one to three days from plating, depending on the cell line growth rate. The drug antiproliferative effects were assessed by cell counting using a Coulter Counter (Beckman Coulter, Luton, UK) 72 h or 96 h later, according to the cell proliferation features (time lag and doubling time) and drug responsiveness reported [53] and assessed in preliminary experiments. Drug concentrations able to inhibit cell growth by 50% (IC_50_) were calculated from dose-response curves.

For drug combination studies, CME-1 cells were simultaneously exposed to the indicated concentrations of SAHA and trametinib. A sequential schedule was used for evaluating the combination of SAHA and trametinib in MoJo, Yamato-SS, Aska-SS and 1273/99 cells using single drug concentrations in the range of the respective IC_25_ and IC_50_ after 72 h of treatment. The interaction of SAHA with trametinib was analyzed according to Chou-Talalay [54] using the Compusyn software 1.0 (www.combosyn.com). By this method, a combination index (CI) value = 1 indicates an additive effect, CI < 1 synergy, and CI > 1 antagonism. Alternatively, the interaction of SAHA in a range of concentrations (0.375-3 μM) with SST0001 at a fixed concentration (0.5 mg/ml) producing alone a negligible antiproliferative effect (about 5% of inhibition) was evaluated by the synergistic ratio index (SRI) as described by Kern et al. [55]. According to this method, SRI > 1 indicates synergy, SR ≤ 1 absence of synergy/additive effect.

For treatment with human active recombinant heparanase (R&D systems, Minneapolis, MN) (Supplementary Table S1), cells plated in complete medium for 24 h were incubated in serum-free medium with or without 5 μg/ml recombinant enzyme for 24 h and 48 h.

### Western blotting

Adherent and floating cells were processed for total protein extraction and Western blotting as previously described in details [56]. Samples from at least two independent experiments were analyzed using antibodies listed in Supplementary Table S1. The densitometric analysis on blots was done using Image J 1.46 R (https://imagej.net).

### Nuclear and cytoplasm fractioning

Cytoplasmic and nuclear cell fractions were prepared using the NE-PER Nuclear and Cytoplasmic Extraction Kit (Thermo Fisher Scientific, Rockford, IL) (Supplementary Table S1) according to the manufacturer’s instructions. Protein fractions were then analyzed by Western blotting.

### Heparanase inhibition assays

SST0762NA1 ability to inhibit heparanase enzymatic activity was assessed using a colorimetric assay measuring the appearance of the disaccharide product of enzyme-catalyzed cleavage of the pentasaccharide fondaparinux (AGA*IA) as previously described in details in [57].

### RNA extraction and quantitative RT-PCR (qRT-PCR)

Total RNA was isolated from control and drug-treated cell lines using RNeasy Plus Mini Kit (Qiagen, Hilden, Germany). Nucleic acid purity and concentration were measured spectrophotometrically using NanoDrop 2000c (Thermo Fisher Scientific). One μg of RNA was reverse transcribed using High Capacity cDNA Reverse Transcription Kit in 20 μl of reaction volume according to the manufacturer’s instructions (Applied Biosystems, Foster City, CA). Amplification of the synthesized cDNA was performed using TaqMan Universal Master Mix (Applied Biosystems). The qPCR assays for *HPSE*, *EGR1,* and *GAPDH* (PrimeTime Integrated DNA Technologies, IDT, Coralville, IA) are reported in Supplementary Table S1. Details of the primers’ sequences were not provided by the Company*. GAPDH* was used as an internal control with minimal expression variations upon different treatments. Amplification reactions, in a final volume of 10 μl, were conducted using the 7900 HT Fast Real-Time PCR System (Applied Biosystems). Each sample was measured in triplicate and qRT-PCR experiments were repeated at least three times. Relative levels of the transcripts of interest were determined using the 2^-ΔΔCt^ method.

### RNA interference

For gene silencing, cells were transfected 24 h after plating with specific or non-targeting siRNAs using Lipofectamine RNAiMAX (Thermo Fisher Scientific) in serum-free medium Opti-MEM I (Invitrogen, Carlsbad, CA). For *HPSE* silencing, 25 nM prevalidated HPSE Silencer Select (s21306/siR06) and the negative control siRNA Silencer Select #2, both from Ambion (Austin, TX), were used. After 5 h, the transfection medium was replaced with complete medium. Knock-down of *SS18-SSX2* was performed using two published and validated *SSX2* specific duplex oligos siRNAs, SSX2A and SSX2B (30 nM) [18, 50]. The non-targeting siRNA Silencer Select #1 (Ambion) was used as negative control. Cells were incubated with the siRNAs for 6 h before addition of serum, and then processed for mRNA and protein extraction 48-72 h later. For *EGR1* silencing, cells were transfected with 60 nM *EGR1* Silencer Select siRNA or the negative control siRNA Silencer Select #2 (Ambion) and serum was added to the medium 6 h later. After 48 h of transfection, cells were exposed to 10 nM FK228 for 6 h and then lysed for mRNA and protein extraction. The suppliers of siRNAs are listed in Supplementary Table S1.

### Apoptosis assays

After drug treatment as indicated, apoptosis was assessed in floating and adherent cells by TUNEL (Terminal deoxynucleotidyl transferase dUTP Nick End Labeling) assay by applying the In Situ Cell Death Detection Kit (Roche, Mannheim, Germany). Sample analyses were performed by the flow cytometer Accuri C6 (BD Biosciences, San Jose CA). Alternatively, apoptosis was assessed photometrically by using the Cell Death Detection ELISA Plus assay (Roche). The cytoplasmic histone-associated DNA fragmentation detected by this assay was corrected for protein content evaluated by Sulforodamine B assay performed in parallel. Data were normalized versus untreated control and expressed as apoptotic index. The Apocyto Caspase 3 colorimetric assay kit (MBL International Sunnyvale, CA) was used to analyze the caspase 3 specific activity according to the manufacturer’s instruction. The suppliers of assays are listed in Supplementary Table S1.

### Immunofluorescence microscopy

For indirect immunofluorescence staining of heparanase, cells were fixed in 3.7% formaldehyde for 15 min and permeabilized in 0.1% Triton X-100 for 5 min. After blocking in 1% BSA in PBS for 1 h, cells were incubated with anti-heparanase antibody (1:50) (Abcam Cambridge, UK) followed by Alexa Fluor 488 anti-rabbit antibody (Thermo Scientific, Rockford, IL) (Supplementary Table S1).

For intracellular detection of the biotin-conjugated SST0762NA1, 48 h after seeding in complete medium, cells were serum starved and treated with the drug (1 mg/ml) for 24 h. Then, cells were fixed with 2% paraformaldehyde and permeabilized in cold methanol for 1 min. After blocking in 1% BSA in PBS for 1 h, cells were incubated with streptavidin Alexa Fluor 488 conjugate (Invitrogen) (Supplementary Table S1). Nuclei were counterstained with Hoechst 33341 (Sigma-Aldrich, St. Louis, MO). Slides, mounted in Mowiol mounting medium (Sigma-Aldrich), were examined by a fluorescence microscope equipped with digital camera.

### In vivo studies

All in vivo experiments were authorized by the Italian Ministry of Health and were performed in accordance with the EU Directive 2010/63/EU for animal experiments, internal institutional guidelines and international policies [58]. Experiments were carried out using female SCID mice (CB17/Icr-*Prkdc*^*scid*^/IcrIcoCrl Charles River, Calco, Italy) housed in cages cleaned regularly with food and water available ad libitum. For experiments, mice were randomized in groups of 6–8 animals, each bearing one tumor xenograft. Mice were monitored daily and tumor growth was monitored at least two times weekly. At the end of experiments, mice were euthanized by cervical dislocation.

Exponentially growing CME-1 cells (20 × 10^6^) were injected orthotopically (i.m.) in the right leg of SCID mice under general anesthesia (100 mg/kg ketamine, 5 mg/kg xilazine i.p.). Treatments started 1 day after tumor cells injection. SAHA, dissolved in 10% DMSO, 5% Cremophor and 85% PBS, was administered by oral gavage at 100 mg/kg, daily, for 5 consecutive days per week, for 4 weeks (qdx5/wx4w). SST0001, dissolved in sterile water, was administered s.c. at 60 mg/kg/injection, twice daily, for 5 consecutive days per week with treatment repeated for 4 weeks (2qdx5/wx4w). Control mice were treated with the SAHA vehicle. Doses and scheduling of drugs were chosen on the bases of previous in vivo studies [51, 59, 60]. Tumor growth was followed by biweekly measurements of tumor diameters with a Vernier caliper. The efficacy of treatments was assessed as tumor volume inhibition percentage (TVI%) calculated according to the formula: TVI% = 100 − (mean TV treated/mean TV control × 100). Drug tolerability was evaluated as body weight loss. Experiments performed with the SUDHL4 and RPMI8226 models are described in Supplementary Material.

### Statistical analyses

The two-tailed Student’s *t*-test was used to compare two sets of data. The Kruskal-Wallis test followed by Dunns post hoc test was used for the comparison among multiple groups. Analyses were performed using the GraphPad Prism software, version 4.0 (GraphPad Prism Inc., San Diego, CA). A two-way ANOVA was applied to test the interaction between treatments and time–course according to the online tool TumGrowth (https://kroemerlab.shinyapps.io/TumGrowth). Holm correction was set for post hoc multiple comparisons. *P* values < 0.05 were considered as statistically significant.

## Results

### HDACi treatment induces variable death response in SS cells

**Fig. 1:**
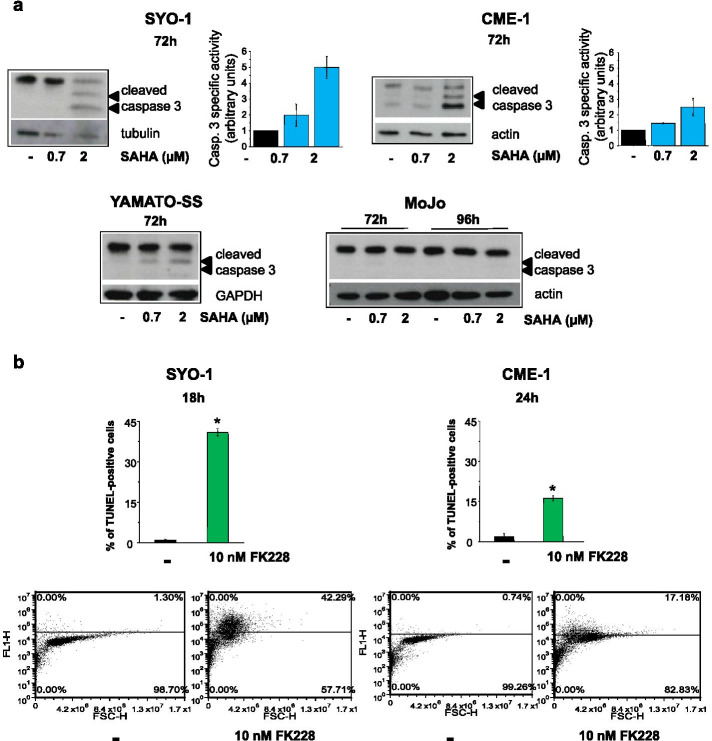
HDACi treatment induces variable death response in SS cells. **a** Caspase 3 activation induced by SAHA was analyzed after 72 h by Western blotting in SYO-1, CME-1 and Yamato-SS cells, or after 72 h and 96 h in MoJo cells. Arrows indicate fragments of activated caspase 3. Actin, tubulin and GAPDH show protein loading. Caspase 3 enzyme activity was measured in SYO-1 and CME-1 cells after 72 h-treatment by a colorimetric assay. Data are reported as arbitrary units ± SE from two independent experiments. **b** SYO-1 and CME-1 cells, exposed to 10 nM FK228 for 18 h and 24 h, respectively, were subjected to apoptosis detection by TUNEL assay. Data are reported as mean % of TUNEL-positive cells ± SE from two independent experiments and representative dot plots of FACS analysis are shown. *P < 0.05

**Fig. 2:**
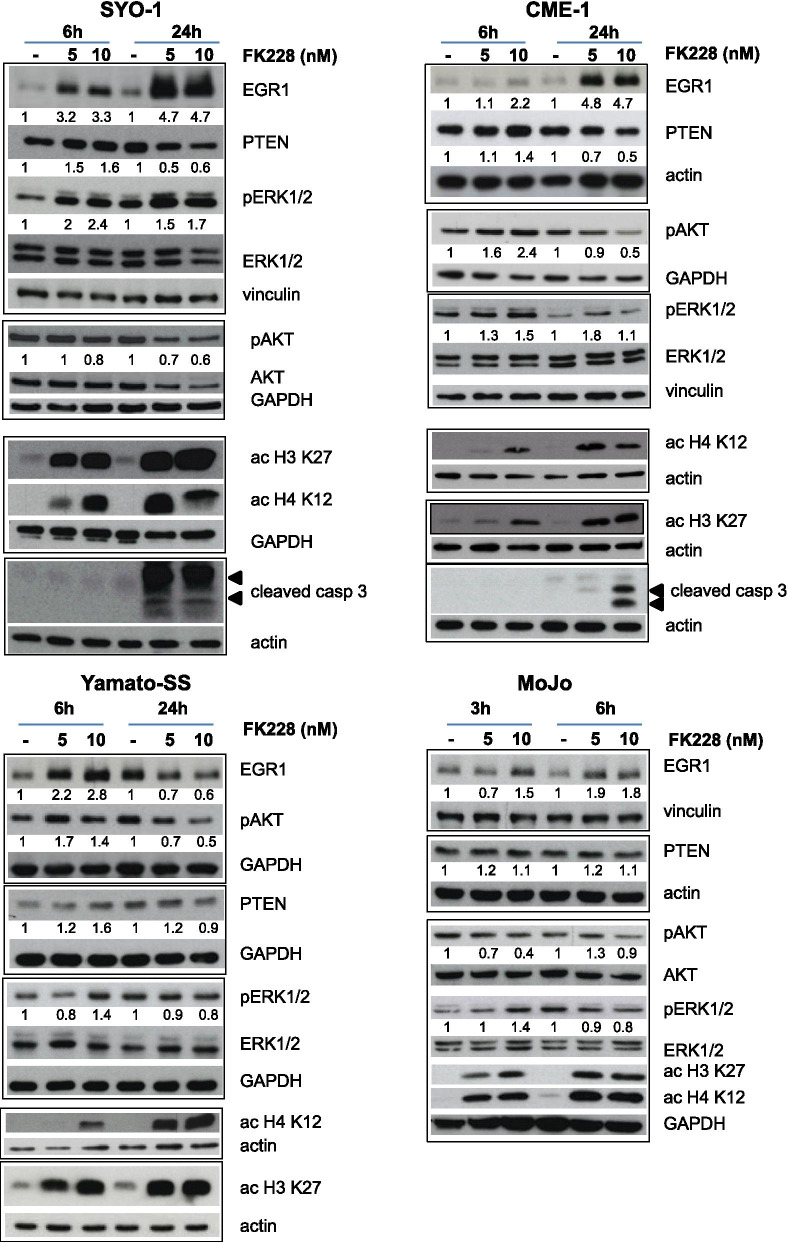
FK228 treatment induces early EGR1 and PTEN upregulation and ERK activation followed by AKT inhibition in SS cells. Cells, exposed to FK228 for the indicated times, were processed for Western blot analysis with the indicated antibodies. Acetylation of histones H3 (K12) and H4 (K27) is shown as marker of HDAC inhibition. Cleavage of caspase 3 evidenced cell death in SYO-1 and CME-1. Arrows indicate fragments of activated caspase 3. Anti-actin, −tubulin, −vinculin and -GAPDH blots show protein loading. Numbers represent the intensity of relevant bands normalized to the respective loading controls

The antiproliferative effect of the two structurally unrelated HDACi SAHA and FK228 [11, 61] was assessed by cell counting in human SS cell lines harboring different SS18-SSX chimeric proteins and various additional genetic alterations (Supplementary Table S2). CME-1, SYO-1 and Yamato-SS cells exhibited a comparable sensitivity to SAHA and FK228 in terms of cell growth inhibition showing similar IC_50,_ whereas MoJo cells appeared slightly less responsive (Table 1). However, the four cell lines underwent different outcomes as evidenced by caspase 3 activation and TUNEL staining. The occurrence of apoptosis upon exposure to either drugs confirmed the high susceptibility of SYO-1 cells to the cytotoxic effect of HDACi as previously reported [19, 21, 22]. To assess if drug-induced growth inhibition in the slowly growing MoJo cells reflected a cytostatic rather than cytotoxic effect, the time exposure to SAHA, less potent than FK228, was extended up to 96 h. MoJo cells appeared refractory to apoptotic cell death as neither caspase 3 cleavage nor TUNEL-positive cells were detected after exposure to SAHA up to 96 h (Table 1, Figs. 1a and S1b). SAHA-treated CME-1 cells displayed reduced caspase activation and TUNEL positivity compared to SYO-1 cells, while incomplete caspase 3 cleavage and no TUNEL staining were observed in drug treated Yamato-SS cells (Table 1, Figs. 1a and S1b). A similar pattern of apoptotic response was observed after exposure to FK228 (Figs. 1b and 2).

**Table 1:** Antiproliferative and proapoptotic activity of HDACi in SS cells

Cell line	SAHA			FK228
	IC_**50**_^**a**^ (μM)	IC_**50**_^**a**^ (μM)	% TUNEL positive	IC_**50**_^**a**^ (nM)
	(72 h)	(96 h)	cells^**b**^	(72 h)
**SYO-1**	0.7 ± 0.1	0.5 ± 0.01	10.5 ± 1	4.4 ± 0.8
**CME-1**	0.8 ± 0.2	0.7 ± 0.2	6.7 ± 0.2	4.2 ± 0.1
**MoJo**	1.4 ± 0.5	0.8 ± 0.2	-^c^	8.4 ± 1.6
**Yamato-SS**	0.5 ± 0.2	0.5 ± 0.2	-^c^	3.8 ± 0.7

^a^ IC_50_, drug concentration inducing 50% inhibition of cell growth after the indicated time of exposure to the drug. IC_50_ values are expressed as mean ± SE from at least two biological replicates performed in duplicate. Raw data are reported in Supplementary Table S3

^**b**^ Apoptosis was assessed by TUNEL assay after drug treatment (2 μM) for 72 h in SYO-1, CME-1 and Yamato-SS cells and 96 h in MoJo cells. Mean percent of positive cells ± SE from at least two biological replicates are reported

^c -^ no TUNEL staining detected in drug-treated cells

Overall, these findings indicated that the growth inhibitory activity exerted by HDACi was not indicative of cell death.

### Targeting HDACi-induced activation of ERK-EGR1 pathway by MEK inhibition results in synergistic cell growth inhibition

Exploration of druggable effectors associated with response to HDACi might provide a means to enhance apoptosis induced by HDACi in SS cells. Therefore, we first analyzed the effects of drug treatments on the activation of survival pathways such as AKT- and ERK-mediated signaling which have been implicated in cell responsiveness to HDACi [12, 19, 62–66]. Specifically, in SS cells, an EGR1-PTEN network has been found reactivated by HDACi [19, 21, 22] which, by disrupting the repressive control exerted by SS18-SSXs, re-establishes the transcription of *EGR1* (HGNC ID 3238), a crucial transcription factor and positive regulator of *PTEN* (HGNC ID 9588). Upregulation of the PTEN phosphatase, and the consequent inhibition of the PI3K/AKT pathway, have been proposed to contribute, in turn, to HDACi-induced apoptosis in SS cells [19]. Consistently with the reported studies, an effective inhibition of HDAC activity in FK228-treated cells, confirmed by a marked increase of acetylation of H3 and H4 histones, was associated with upregulation of EGR1 which appeared earlier in MoJo cells (Fig. 2). Inhibition of AKT phosphorylation was also observed in treated cells in the face of a transient/low upregulation of PTEN (Fig. 2). Notably, a direct inhibition of PI3K enzyme activity by FK228 [61] might also contribute to downregulate AKT phosphorylation.

**Fig. 3:**
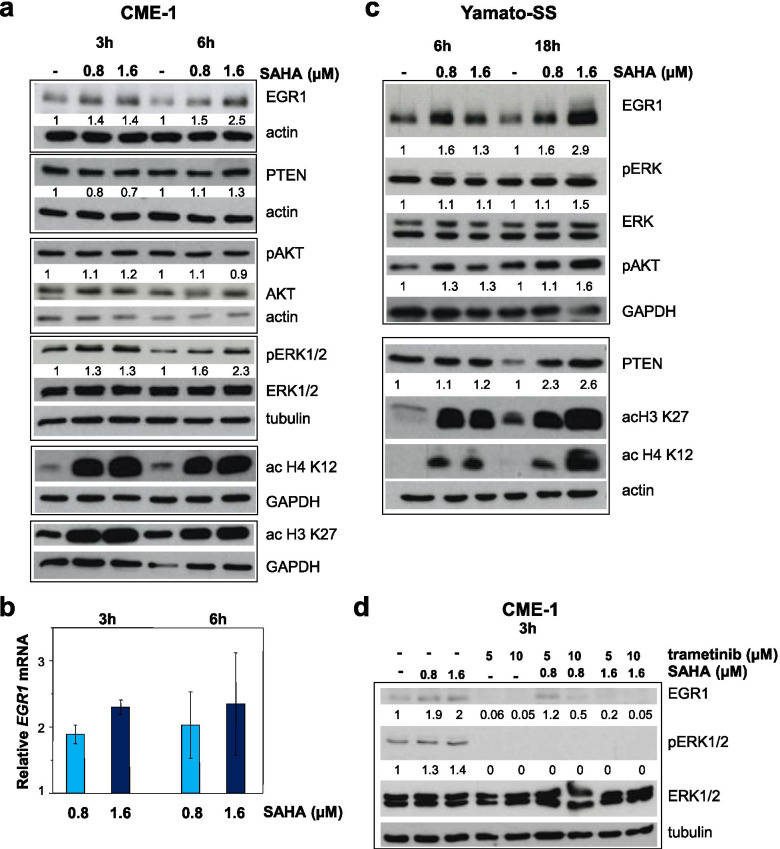
SAHA treatment induces ERK-dependent upregulation of EGR1. **a**, **b** and **c** CME-1 and Yamato-SS cells were exposed to SAHA for the indicated times. In (**a**) and (**c**) cells were processed for Western blot analysis with the indicated antibodies. Acetylation of H3 and H4 at K27 and K12, respectively, is shown as marker of HDAC inhibition. In (**b**) *EGR1* mRNA expression was analyzed by qRT-PCR and expressed as relative quantification with respect to untreated cells as calibration sample. Mean relative mRNA values ± SE from three independent experiments are reported. **d** Effect of 3 h-treatment with SAHA and trametinib, alone and in combination, on ERK activation and EGR1 expression assessed in CME-1 cells by Western blotting. In (**a**), (**c**) and (**d**) actin, tubulin and GAPDH show loading control. Numbers represent the intensity of relevant bands normalized to the respective loading controls

The uncoupling between the drug-induced modulation of the EGR1-PTEN and -AKT inhibition was more evident in SS cells exposed to SAHA (Fig. 3). Treatment of CME-1 cells with SAHA induced a marked increase of histone acetylation already evident after 3–6 h, which clearly paralleled an increase of EGR1 at both mRNA and protein level (Fig. 3a and b). However, drug-induced upregulation of the transcription factor and PTEN modulation were not associated with inhibition of AKT phosphorylation in both CME-1 and Yamato-SS cells (Fig. 3a and c). Instead, an increased AKT phosphorylation was observed in Yamato-SS cells (Fig. 3c) possibly related to aberrant pathway activation due to *PIK3CA* (HGNC gene ID 8975) mutation (Supplementary Table S2). These findings suggested that modulation of the PTEN/AKT pathway does not necessarily reflect an apoptotic response to HDACi in SS cells.

Contrasting with inconsistent effects of the two HDACi on PTEN-AKT signaling, activation of the ERKs paralleled EGR1 upregulation in cells treated with either FK228 or SAHA (Figs. 2, 3a and c). A constitutive ERK pathway activation has been correlated to high EGR1 expression in several tumor cells [67, 68]. Accordingly, higher levels of EGR1 were observed in SS cells exhibiting enhanced ERK phosphorylation (Supplementary Fig. S1c), thus suggesting a key regulatory role for this pathway. As constitutive or drug-induced ERK activation could counteract the cytotoxic effect of HDAC inhibition, we examined the effects of the MEK1 inhibitor trametinib on the ERK-EGR1 cross-talk in SS cells. As shown in Fig. 3d, trametinib abrogated ERK phosphorylation and prevented the enhancement of EGR1 expression induced by SAHA in CME-1 cells exposed to the drug combination. This finding supported the upstream role of the ERK pathway activation in HDACi-induced EGR1 upregulation. The combined treatment produced a synergistic antiproliferative effect and enhanced the apoptotic response (Supplementary Fig. S2a). A synergistic interaction between the two drugs was also observed in MoJo cells harboring the *NRAS* (HGNC gene ID 7989) Q61R mutation and constitutive ERK activation [68]. The most effective interaction between the two drugs was recorded in these cells upon 24 h pretreatment with SAHA followed by exposure to trametinib at concentrations spanning the IC_50_ at 72 h [68] (Supplementary Fig. S2b). These treatments promoted an apoptotic response in MoJo cells (Supplementary Fig. S2b). A synergistic antiproliferative effect was also observed in Yamato-SS, ASKA-SS and 1273/99 cells treated with the sequential combination schedule (Supplementary Fig. S2c).

These findings revealed a relevant contribution of the ERK pathway to the expression of EGR1 in SS cells. Activation of this cytoprotective pathway by HDACi can be counteracted by a MEK inhibitor eventually resulting in a positive drug interaction and possibly apoptosis induction in cells harboring different genetic background (i.e. *SS18-SSX* translocation) and further alterations causing ERK activation.

### HDACi upregulate heparanase through EGR1 induction in a self-sustainig circuit

**Fig. 4:**
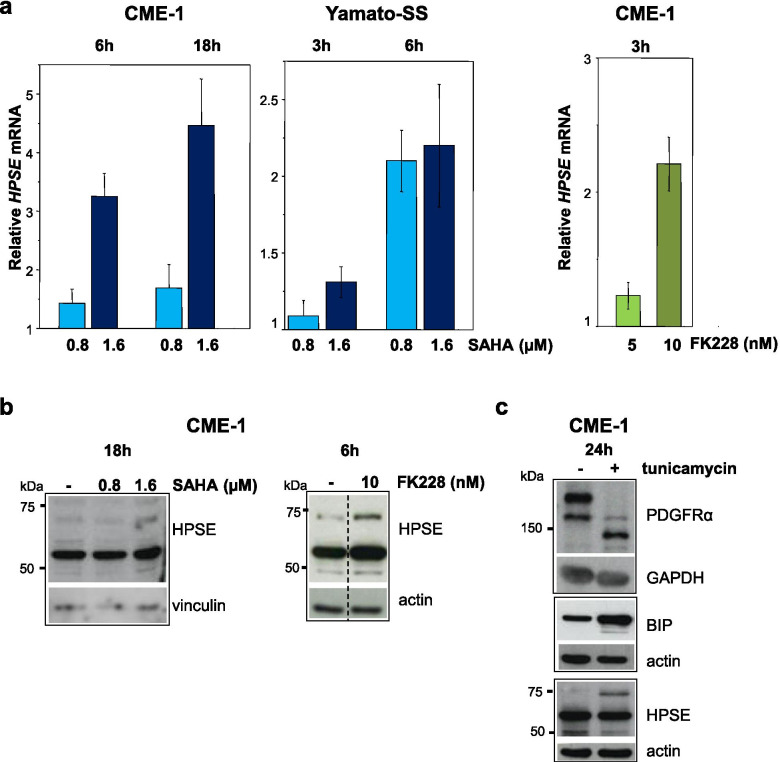
HDACi upregulate heparanase mRNA and protein. **a** Analysis of *HPSE* expression by qRT-PCR in SS cells treated with SAHA or FK228 for the indicated times. Data are reported as relative quantification with respect to untreated cells as calibration sample. Relative mRNA values are the mean ± SE from three independent experiments. **b** Western blot detection of heparanase polypeptides in CME-1 cells exposed to SAHA or FK228 for the indicated times. Image on the right is from a cropped blot (dashed line) from which lanes not of interest have been removed. **c** N-glycosylation inhibition does not modify electrophoretic mobility of heparanase polypeptides. CME-1 cells were treated with tunicamycin (2 μg/ml for 24 h) and processed for Western blotting. As controls, the shift of PDGFRα bands shows the glycosylation inhibition and BIP upregulation indicates endoplasmic reticulum stress. In (**b**) and (**c**) anti-actin, −vinculin and -GAPDH blots are shown as loading control

To identify additional druggable targets to improve the therapeutic response to HDACi in SS, we examined whether EGR1 upregulation modulated the expression of the endo-β-D-glycosidase heparanase. In fact, the *HPSE* gene has been demonstrated to be variably regulated at transcriptional level by EGR1 depending on the cellular context [69–76]. Specifically, the ERK-EGR1 pathway has been implicated in the inducible transcription of *HPSE* [69, 75]. In line with ERK activation and EGR1 upregulation (Figs. 2, 3a and c), a time- and dose-dependent increase in *HPSE* transcription was observed in cells treated with SAHA and FK228 (Figs. 4a and S3a). At protein level, western blot analysis showed the upregulation of three heparanase polypeptides in cells exposed to the HDACi: two bands of 65 kDa and 50 kDa corresponding to the proform and the active enzyme, respectively, and an additional band of about 70 kDa (Fig. 4b). Because the latter band appeared as the most upregulated by treatment, we further investigated the nature of the high molecular weight form of heparanase. CME-1 cells were treated with the glycosylation inhibitor tunicamycin as N-glycosylation has been shown to modify the electrophoretic mobility of the protein [77–79]. As controls, the mobility shift of PDGFRα isoforms confirmed the N-linked oligosaccharide removal [80] while the upregulation of the endoplasmic reticulum (ER) chaperone BIP indicated ER stress and impairment of intracellular trafficking in cells treated with the antibiotic [81]. Tunicamycin did not produce a size reduction of the high molecular weight heparanase form but, on the contrary, enhanced its level (Fig. 4c). Since inhibition of N-glycosylation has been shown to affect the kinetics of ER to Golgi transport and secretion of heparanase [77], we hypothesize that the high molecular weight form represents the pre-proheparanase, described as a protein of about 68 kDa [78], hindered from ER targeting following tunicamycin treatment. These findings suggested an effect of HDAC inhibition on inducible transcription of heparanase.

**Fig. 5:**
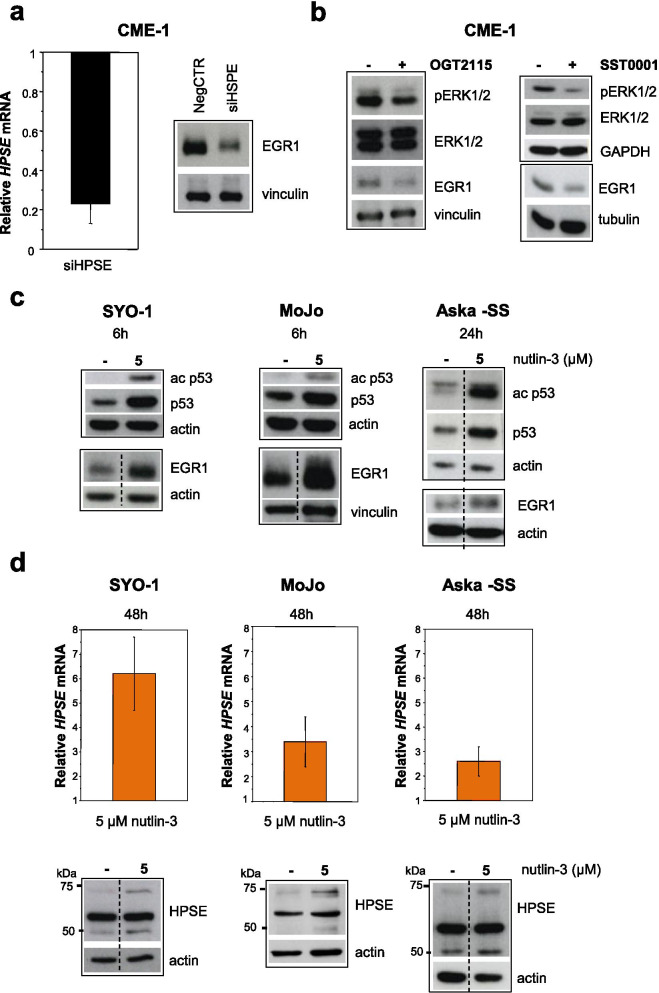
ERK-EGR1 are positively regulated by *HPSE* and wt p53 acetylation/stabilization is associated with upregulation of EGR1 and *HPSE* expression in SS cells. **a, b** *HPSE* silencing and pharmacological blockade inhibit the ERK-EGR1 axis. In (**a**), 72 h after transfection with aspecific RNA oligonucleotide (NegCTR) or *HPSE* siRNA (siHPSE), CME-1 cells were processed for mRNA and protein extraction. On the left, *HPSE* knock-down was assessed by qRT-PCR. The mean relative quantification value ± SE with respect to NegCTR samples from two independent experiments is reported. On the right, EGR1 protein levels were analyzed in whole cell lysates by Western blotting. In (**b**) CME-1 cells were exposed to 0.5 μM OGT2115 for 48 h or 0.5 mg/ml SST0001 for 18 h. Effects of drug treatments on ERK activation and EGR1 expression were assessed in whole cell lysates by Western blotting. Vinculin, GAPDH and tubulin are shown as controls for protein loading. **c** SYO-1, MoJo and ASKA-SS cells, harboring wt *TP53*, were exposed to 5 µM nutlin-3 for the indicated times and then processed for Western blotting to detect p53 acetylation at K382 and levels of p53 and EGR1 proteins. **d** After 48 h- treatment with nutlin-3, *HPSE* mRNA and protein levels were assessed in SS cells by qRT-PCR and Western blotting. Relative quantification with respect to untreated cells as calibration sample is reported. Mean relative mRNA values ± SE from at least three independent experiments are shown. Actin and vinculin are shown as loading controls in immunoblots. Dashed lines indicate cropping in blots from which lanes not of interest have been removed

Because previous studies suggested a reciprocal regulation of EGR transcription factors and heparanase expression [75, 82], we examined the potential role of heparanase in regulating EGR1 expression in SS cells. *HPSE* gene silencing by RNA interference confirmed the impact of heparanase on EGR1 expression in CME-1 cells inducing downregulation of the transcription factor (Fig. 5a). A similar effect was observed in cells treated with OGT2115, a small molecule heparanase inhibitor [83], and SST0001, a HS mimetic/heparanase inhibitor currently under clinical investigation [52] (Fig. 5b). Notably, the two drugs also inhibited ERK activation suggesting a functional requirement for the β-endoglycosidase enzyme activity in regulating EGR1 and confirming the presence of an ERK/EGR1/heparanase self-sustaining circuit.

### wt-p53 acetylation contributes to HDACi-induced heparanase upregulation

Transcription of the *HPSE* gene is controlled through various mechanisms involving both activating factors (e.g. EGR1) and negative regulators (e.g. p53). Deregulation of these players, and additional levels of control at RNA and protein level, collectively contribute to the increased heparanase expression in human tumors [79]. A direct binding of the tumor suppressor p53 to the *HPSE* promoter involving the recruitment of HDACs has been previously demonstrated and histone deacetylation proposed as mechanism of negative regulation of *HPSE* expression by p53. This regulatory function is lost by several *TP53* (HGNC gene ID 11998) mutants and treatment with HDACi abolished the transcriptional repression of *HPSE* by the wt tumor suppressor [84]. On the other hand, p53 acetylation and the consequent increased protein stability can be a consequence of HDAC inhibition [85]. In fact, treatment with FK228 of SYO-1 and MoJo cells harboring wt *TP53* (Supplementary Table S2) rapidly induced acetylation of p53 along with its stabilization (Supplementary Fig. S3b). Since a reciprocal interaction between p53 and EGR1 has been described [67], we asked whether p53 acetylation/stabilization induced by HDACi contributed as an additional mechanism promoting heparanase expression. Indeed, overexpression of wt *TP53* in senescent or doxorubicin-treated endothelial cells has been recently related to upregulation of both EGR1 and heparanase [86]. As an alternative way to induce p53 acetylation/stabilization, we treated SYO-1, MoJo and Aska-SS cells harboring wt *TP53* (Supplementary Table S2) with nutlin-3. This MDM2-p53 binding inhibitor, by displacing p53 from MDM2, enhances p53 acetylation and its stability [69, 87, 88]. In fact, the increased expression of an acetylated p53 observed in cells treated with nutlin-3 was accompanied with EGR1 upregulation followed by heparanase upregulation at both mRNA and protein level (Fig. 5c and d). Conversely, p53 acetylation/stabilization and *HPSE* or *EGR1* expression were not affected by nutlin-3 in p53-mutant Yamato-SS cells (Supplementary Table S2 and Supplementary Fig. S3c). These effects indicated loss of the negative control by an acetylated functional p53 on heparanase expression.

**Fig. 6:**
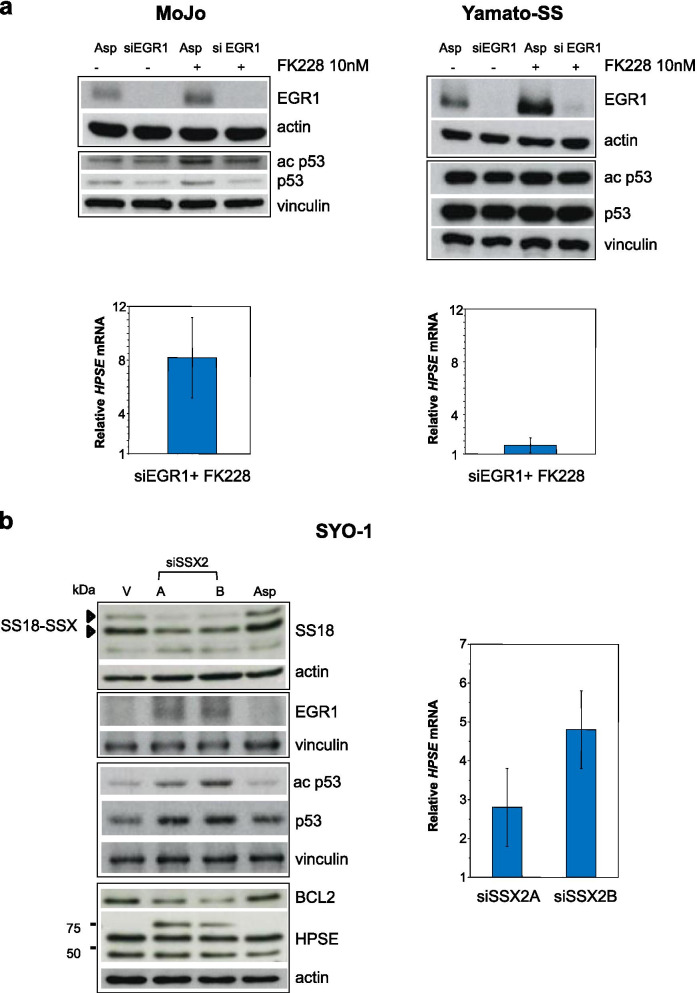
EGR1 and p53 cooperate to regulate *HPSE* expression in SS cells in response to HDAC inhibition. **a** *HPSE* is enhanced by FK228 treatment in wt p53 expressing EGR1 silenced cells. After 48 h of transfection with an aspecific RNA oligonucleotide (Asp) or *EGR1* siRNA (siEGR1), MoJo (wt p53) and Yamato-SS (mut p53) cells were exposed to 10 nM FK228 for 6 h and then processed for Western blot analysis of EGR1 and p53 (upper panels) or qRT-PCR analysis of *HPSE* mRNA (lower panels). Mean values from three (MoJo) or two (Yamato-SS) independent experiments are reported. **b** *SS18-SSX2* silencing upregulates EGR1, p53 and heparanase. SYO-1 cells were treated with transfection reagent (V), two specific siRNAs targeting the oncogene (siSSX2A and siSSX2B) or an aspecific RNA oligonucleotide (Asp) at 30 nM final concentration. Seventy two hours after transfection, cells were processed for protein and mRNA analysis. On the left, immuno blotting performed on whole cell lysates with the indicated antibodies. Arrows indicate the fusion oncoproteins. On the right, *HPSE* expression assessed by qRT-PCR. Mean values from three independent experiments are reported. In (**a**) and (**b**) actin and vinculin are shown as protein loading controls. The mean relative *HPSE* quantification values ± SE are referred to aspecific control

To further explore the role of EGR1 and p53 in heparanase induction by HDACi, we silenced *EGR1* in MoJo and Yamato-SS cells harboring wt and mutated p53, respectively (Supplementary Table S2). As shown in Fig. 6a, following *EGR1* knock-down, FK228 substantially induced *HPSE* only in MoJo cells supporting the role of wt p53 acetylation in the upregulation of the endoglycosidase in response to HDACi.

### Heparanase is epigenetically regulated and acts as epigenetic regulator in SS cells

Because HDACi can affect gene expression regulated by HDAC recruited by the fusion oncoproteins and the complex with SS18-SSX has been implicated in the repressive control of *EGR1* in SS cells [9, 14–17, 20], we knocked down the fusion gene to investigate whether the fusion protein was involved in the regulation of heparanase expression. Similarly to what observed by direct HDAC inhibition (Figs. 2 and S3a), the upregulation of EGR1 in SYO-1 cells transfected with *SS18-SSX2* siRNAs was associated with an increase in heparanase expression suggesting a role for the oncoprotein in the control of the EGR1-heparanase axis mediated by HDAC (Fig. 6b). Moreover, accordingly to previous data (Figs. 5c and S3b), the reduced expression of the SS18-SSX2-HDAC complex also promoted acetylation and stabilization of p53. As expected [89, 90], the oncogene knock-down was also associated with a reduced expression of the anti-apoptotic protein Bcl-2 (Fig. 6b). In CME-1 cells, *SS18-SSX2* silencing upregulated the expression of both EGR1 and heparanase whereas, as expected, the dysfunctional p53 expressed in these cells was not affected (Supplementary Fig. S3d).

**Fig. 7:**
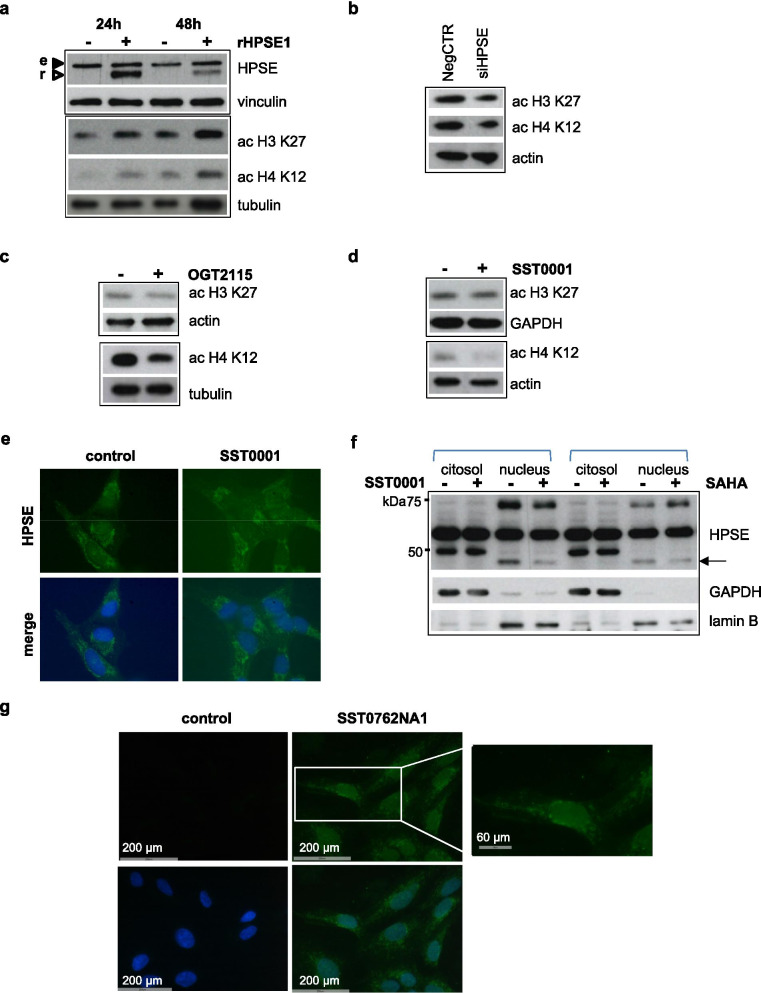
Heparanase promotes histone acetylation and its inhibition reduces nuclear localization. **a** Serum starved CME-1 cells were incubated with human active recombinant heparanase (5 μg/ml) for the indicated times. Then, cells were lysed and processed for immunoblotting with the specified antibodies to detect heparanase (r, recombinant 50 kDa heparanase; e, endogenous 65 kDa heparanase) and acetylation of H3 (K27) and H4 (K12). Histone acetylation was also analyzed in cell lysates after transfection with aspecific RNA oligonucleotide (NegCTR) or *HPSE* siRNA for 72 h **(b)** and in cells treated with OGT2115 (0.5 μM) for 48 h or SST0001 (0.5 mg/ml) for 24 h **(c)**. Vinculin, GAPDH and tubulin are shown as controls for protein loading. **d** Indirect immunofluorescence showing localization of heparanase in control and SST0001-treated (1 mg/ml for 18 h) cells. Nuclei are evidenced with Hoechst 3341 counterstaining (blue). Original magnification, 1000X. **e** Cytoplasmic and nuclear fractions from cells exposed to 0.5 mg/ml SST0001 or 1.6 μM SAHA for 18 h, were analyzed by Western blotting to examine intracellular distribution of heparanase polypeptides. Lamin B and GAPDH are shown as controls for nuclear-cytoplasmic fractioning and loading. **f** Biotin-conjugated SST0001 analogue SST0762NA1 enters the nuclei. Serum starved cells treated with SST0762NA1 (1 mg/ml) for 24 h were fixed, permeabilized, and incubated with streptavidin Alexa Fluor 488 conjugate to detect the drug. Cells were stained with Hoechst 33341 to evidence nuclei. Inset, enlarged detail evidencing SST0762NA1 localization in the nucleus and in perinuclear vesicles

Accumulating evidence suggests a role for heparanase in regulating gene transcription also through epigenetic mechanisms [30, 79, 91–97]. Nuclear localization of heparanase, documented in several studies [34, 35, 96, 97] has been associated with increased histone acetylation due to degradation of nuclear HS which acts as inhibitor of HATs [34, 35]. To explore the role of the heparanase/HPSG axis in epigenetic regulation in SS cells, we first examined the effect of heparanase on histone acetylation. Treatment of CME-1 cells with enzymatically active recombinant heparanase time-dependently increased acetylation of H3 and H4 histones (Fig. 7a). On the other hand, either *HPSE* silencing or treatment with OGT2115 or SST0001 induced a reduction of histone acetylation (Fig. 7b-d). We further examined whether SST0001 treatment influenced heparanase cellular localization. Actually, immunofluorescence evidenced both perinuclear and nuclear localization of heparanase in CME-1 cells, whereas a reduced presence of the enzyme in the nucleus was observed in SST0001-treated cells (Fig. 7e). Western blot analysis of nucleus/cytoplasm-fractionated cells after drug treatment showed no modulation of levels of the 65 kDa heparanase pro-form, present in both fractions, and the 50 kDa active form segregated in the cytoplasm. Unexpectedly, only the high molecular weight form and a small peptide (< 50 kDa), which were mostly detected in the nucleus, appeared reduced by SST0001 treatment (Fig. 7f). As the presence of active heparanase has been described in nuclei of other cells, one possibility might be that the smallest peptide is an active heparanase cleaved by a protease different from that functioning in the cytoplasm [33]. An opposite effect was observed in SAHA-treated cells on the high molecular weight heparanase which showed enhanced levels, whereas the 65 kDa and 50 kDa peptides were not affected by treatment, and the smallest peptide was slightly reduced (Fig. 7f). These findings indicated that SST0001 effects in the nucleus involve different products of heparanase processing and the same products are affected by SAHA.

Based on the above findings and findings by others [34, 35] indicating that the nuclear effects of heparanase could be affected by HS mimetic/heparanase inhibitors such as SST0001, we examined whether these compounds could exert their effects directly in the nucleus. We took advantage of availability of recently produced biotin-conjugated SST0001 derivatives maintaining heparanase inhibitory activity [40]. We used SST0762NA1, a structural analogue of SST0001 characterized by biotin conjugation at glucosamine residues and heparanase inhibitory activity in the same nanomolar range (IC_50_ = 5.93 nM vs 2.08 nM). Fluorescence microscopy of CME-1 cells exposed to SST0762NA1 evidenced a prevalent nuclear localization of the drug along with positivity in perinuclear vesicles (Fig. 7g) reminiscent of heparanase cellular distribution (Fig. 7e). These observations supported the potential of heparin derivatives to affect heparanase activity and interfere with HS-HSPG functions in various cellular compartments including the nucleus.

Overall, these findings supported that the SS18-SSX-HDAC complex regulate heparanase expression. The heparanase/HSPG system, in turn, promotes histone acetylation potentially affecting gene transcription, a function enhanced by HDACi and counteracted by different classes of heparanase inhibitors in SS cells.

### HDACi cooperate with the HS mimetic/heparanase inhibitor SST0001 to inhibit SS growth

Overall, the above observations prompted us to test in vivo the combined inhibition of HDACs and heparanase. Actually, in the context of unpublished previous studies, we found this approach effective and well-tolerated in hematological tumor models treated with SST0001 and SST3595, a hydroxamate-based HDAC inhibitor [51]. The human multiple myeloma RPMI8226 and the lymphoma SUDHL4 xenografts, which express heparanase [35, 98] showed low sensitivity to the HDAC inhibitor alone. SST0001 remarkably enhanced the antitumor efficacy in both tumor models. Indeed, RPMI8226 tumors showed a growth delay under treatment with SST0001 which was further enhanced at each time point in combination with ST3595, although not reaching significance (max TVI 24, 61 and 85% for SST3595, SST0001, and the combination, respectively) (Supplementary Fig. S4). The growth of SUDHL4 tumors was prevented by SST0001 and the combination with ST3595 was highly effective inducing growth regressions with complete responses during treatment in 83% of mice (Supplementary Fig. S4 and Supplementary Table S4).

**Fig. 8:**
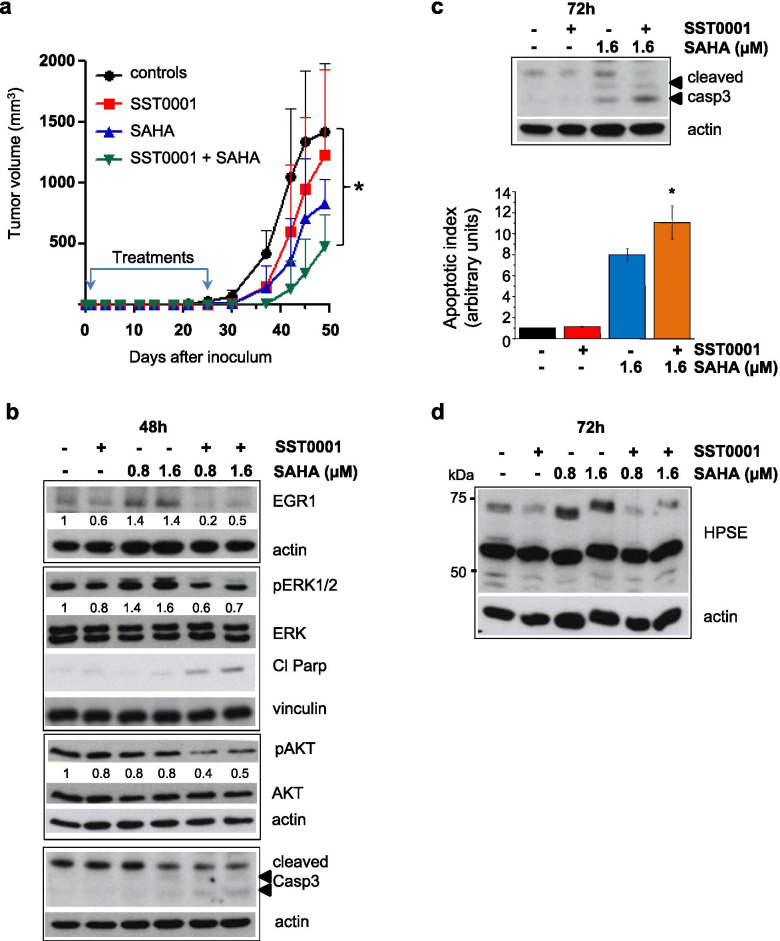
SS tumor growth inhibition and apoptotic cell death are enhanced by co-treatment with SAHA and SST0001. **a** Growth curves of CME-1 xenografts grown in the leg muscle of mice. Animals were treated with vehicle (controls), or SST0001 s.c. at 60 mg/kg, 2qdx5/w for 4 weeks, or SAHA by oral gavage at 100 mg/kg, qdx5/w for 4 weeks, or with the two drugs in combination, starting 1 day after tumor cell injection. Each point is the mean tumor volume in 6/8 mice ± SD. *P < 0.05 referred to the entire curves and the last time point. **b, d** CME-1 cells were treated with SAHA (0.8 and 1.6 μM) and SST0001 (0.5 mg/ml) alone or in combination at the indicated times. The effect of treatments on ERK and AKT activation, EGR1 expression, caspase 3 and PARP cleavage (**b**) and on heparanase expression (**d**) was analyzed by Western blotting. Vinculin and actin are shown as loading controls in immunoblots. Numbers represent the intensity of relevant bands normalized with respect to the respective loading controls. **c** Cells were exposed to SAHA (1.6 μM) and SST0001 (0.5 mg/ml), alone or in combination for 72 h, to detect caspase 3 cleavage by Western blotting and apoptosis by cytoplasmic histone-associated DNA fragmentation assay. Bars represent mean values referred to control cells ± SE obtained in four independent biological replicates. *P < 0.05 vs single agents and controls

Heparanase has been shown to be an actionable target in various sarcoma models [28, 29] and we have reported that both heparin derivatives and small molecule heparanase inhibitors produce a remarkable impact on SS cell malignant behavior in vitro and in vivo [37–39, 60]. To study the effect of the combination of SAHA and SST0001 in a SS model, we used the orthotopic CME-1 tumor xenograft growing in mice. The drugs were administered 24 h after i.m. cell injection for 4 weeks, before the appearance of the tumors, a situation mimicking treatment of minimal residual disease. Under these experimental conditions, at the end of experiment (day 49), 24 days after treatment interruption, SAHA achieved a 42% TVI and SST0001 marginally affected the tumor growth (TVI = 14%). The drug combination prevented the tumor growth for the first 12 days after treatment end. The growth delay by the combination was maintained up to experiment end although significantly enhanced only compared to controls (TVI% = 66%, *P* < 0.05) (Fig. 8a and Table 2).

**Table 2:** Antitumor effects of SAHA and ST0001 against the human synovial sarcoma CME-1

Drug	Dose (mg/kg/day)	Schedule	TVI%^**a**^ (day 49)
**SAHA**	100	qdx5/w x4w	42
**ST0001**	60	2qdx5/w x4w	14
**SAHA + ST0001**	100 60	qdx5/w x4w 2qdx5/w x4w	66*

*P < 0.05 vs control

^**a**^ Tumor Volume Inhibition percent at the end of experiment

When tested in cell culture, SST0001 only slightly inhibited CME-1 cell proliferation, whereas in combination with SAHA produced an additive/synergistic antiproliferative effect (not shown) as indicated by the synergistic ratio index (SRI) calculated by the Kern method (0.9 < SRI < 1.4) [55]. Cells treated with SAHA and SST0001 were characterized by cell death confirmed by cleavage of caspase 3 and PARP already evident after 48 h (Fig. 8b). Caspase 3 cleavage appeared further increased, and apoptosis significantly enhanced, at 72 h (Fig. 8c). The proapoptotic effect of the drug combination was associated with reduced AKT activating phosphorylation. Importantly, the combination prevented the ERK/EGR1 upregulation induced by the HDACi and counteracted the heparanase increase as shown in Fig. 8b and d. These data supported the ability of SST0001 to disrupt the reciprocal positive regulation among components of the ERK-EGR1-heparanase axis stimulated by HDACi in SS cells, thus promoting a proapoptotic effect and enhancing antitumor efficacy.

## Discussion

As a predominantly epigenetic disease, SS is considered epigenetically vulnerable [2, 8]. However, as in other contexts, the cellular consequences of gene epigenetic alteration and modulation of acetylation homeostasis induced by HDACi in SS remain incompletely understood. Compared to the efficacy demonstrated by HDACi in hematological malignancies leading to their clinical approval, results in solid tumors have been disappointing, likely due to poor pharmacokinetic profiles of some of these agents and occurrence of resistance mechanisms [11]. Despite HDACi may reactivate the expression of tumor suppressor genes and modulate genes promoting antitumor effects, their pleiotropic action may not be totally beneficial [12, 24, 99]. Our study evidenced a detrimental upregulation of the ERK/EGR1/heparanase axis induced by HDACi in SS models and provided the rationale for designing drug combinations to improve the cellular response to these agents. Indeed, by intercepting the activation of this pathway, MEK1/2 and heparanase inhibitors enhanced HDACi antiproliferative and proapototic effects. Furthemore, interruption of the EGR1-heparanase self-sustaining circuit by targeting the endo-β-D-endoglycosidase interfered with heparanase-mediated epigenetic effects resulting in reduced histone acetylation. In vivo, the combination of the HS mimetic/heparanase inhibitor SST0001 with HDACi significantly improved the antitumor efficacy in the orthotopic SS xenograft CME-1. A positive interaction between HDACi and SST0001 was also observed in hematologic tumor models.

ERK signaling overactivation plays a key role in counteracting cell response to HDACi and synergistic inhibitory effects between HDACi and agents targeting the ERK pathway have been demonstrated in both hematological and solid tumor models [12, 63, 66, 100, 101]. Although aberrant activation of ERK signaling has been described as a common feature in SS [66, 68, 102–106], only a few studies investigated the role of this pathway in SS cell responsiveness to anticancer drugs. Remarkably, in SS preclinical models, ERK overactivation through diverse mechanisms, such as MKP3 downregulation [107] or *NRAS* Q61R mutation and deregulated receptor tyrosine kinase activation [68] has been shown to play a relevant role in intrinsic and acquired resistance to the clinically approved drug pazopanib. Our present results demonstrated a prosurvival role for drug-induced ERK activation in SS cells and provided evidence for a positive interaction between SAHA and the ERK pathway inhibitor trametinib in reducing cell proliferation and inducing apoptosis even in the presence of *NRAS* activating mutation. The translational potential of this combination is strengthened by the growing identification of oncogenic mutations in *HRAS*, *KRAS* (HGNC IDs 5173, 6407), *NRAS* and *BRAF* (HGNC ID 1097) genes in SS cell lines and clinical tumor subsets [68, 102, 103, 106]. Notably, in a case report, Watanabe et al. [106] described a *BRAF* V600E mutation in SS of a patient experiencing tumor shrinkage after treatment with trametinib and the BRAF inhibitor dabrafenib. The local recurrence developed afterward presented an additional *NRAS* Q61K mutation. The occurrence of these oncogenic mutations in SS, a tumor considered mutationally quiet [3, 108], corroborated the nodal role for ERK pathway hyperactivation in promoting tumor growth and progression as well as its therapeutic interest.

In several physiological and pathological conditions, ERK activation induces expression of the EGR1 transcription factor, promoting cell survival or cell death depending on stimulus or insult [67, 68, 109–111]. In the SS cell lines used in this study, a high basal expression of EGR1 was associated with overactivation of the ERKs and our data evidenced a causal relationship between ERK and EGR1 upregulation upon exposure to HDACi. *EGR1*, found downregulated in SS specimens and SS18-SSX inducible models, has been suggested as a putative tumor suppressor in this context [18, 22, 112–115]. Repressive histone modifications and recruitment of PRC to the *EGR1* promoter mediated by SS18-SSX, as well as posttranscriptional regulation by miR183, have been suggested to work in concert to downregulate *EGR1* in SS [18, 116]. By disruption of the repressive control exerted by SS18-SSX-containing chromatin remodeling complexes, HDACi have been proposed to reactivate an EGR1-PTEN pathway promoting AKT inhibition and SS cell death [19, 21, 22]. Although our data confirm EGR1 and PTEN induction by structurally different HDACi, they also evidenced that association with cell death is not univocal. Under our experimental conditions, we observed a transcriptional upregulation of PTEN as previously reported [19] only in SYO-1 cells. Notably, other epigenetic and post-transcriptional mechanims including acetylation of PTEN induced by HDACi treatment [117, 118] might be implicated in the FK228-induced upregulation of the phosphatase at protein level. Our findings supported the induction of EGR1 as a stress-related and tumor defensive mechanism also mediating pro-survival/pro-metastatic signals. In fact, independently of EGR1 basal levels and kinetics of induction by HDACi, the transcription factor upregulation was associated with increased expression of heparanase, a pleiotropic player in tumorigenesis and disease progression [27–32]. In keeping with observations in other contexts [75, 82], we demonstrated that heparanase, in turn, sustained EGR1 expression in a pathologic loop resembling networks/circuits implicated in self-renewal, differentiation and developmental programs, and likely recruited by SS cells to promote escape from HDAC inhibition. In line with these findings, a previous report by Laporte et al. showed upregulation of genes in “extracellular matrix organization”, “positive regulation of MAPK cascade” and “cellular response to stress” along with “regulation of nervous system development” and “neuron differentiation” enriched categories induced in SS cellular models upon exposure to the second generation HDACi quisinostat [21]. Furthermore, a comprehensive functional analysis of EGR1 targets revealed, among others, the enrichment of pathways related to intracellular signaling cascade controlling EGR1 expression itself (e.g. RAS and ERK) and proteoglycans in cancer [109]. Despite the therapeutic relevance of HDACi, a cautionary note on the use of these agents in the clinic has been raised by studies demonstrating a putative tumor suppressor role for some HDACs in certain cellular setting [11, 24, 119, 120] and evidence of epigenetic activation of metastatic and stemness potential in preclinical studies [12, 120, 121]. For instance, in multiple solid tumor models, structurally different HDACi (including SAHA) have been shown to induce cell death and simultaneously activate tumor-progressive genes, such as MMPs, promoting cell migration in vitro and metastatic dissemination in vivo [121]. Notably, MMPs and heparanase induction by HDACi may functionally result in alteration of the extracellular matrix structure and a tumor microenvironment permissive for cell invasion. Moreover, other extracellular matrix degrading proteases may join to this dangerous cooperation as EGR1 is also involved in upregulation of cathepsins including cathepsin L thought to be responsible for heparanase processing and activation [27, 122, 123].

*HPSE* is listed among genes variably regulated by EGR1 depending on the cellular contexts [69–76]. The ERK-EGR1 pathway, in particular, has been implicated in the inducible transcription of heparanase [69, 75]. EGR1, which is recognized as a relevant “conductor for tissue repair orchestra” [110], is functionally interconnected with heparanase in both physiological (e.g. development, vascularization) and pathological (e.g. fibrosis, diabetic nephropathy, vascular proliferative disorders, cancer) conditions involving extracellular matrix remodeling, angiogenesis and cell mobilization [30, 97, 111, 122]. An intriguing finding in our study was that enhancing effects of HDACi on EGR1 and heparanase were mimicked by *SS18-SSX2* RNA interference in SS cells, in line with the assumption that HDAC is a core subunit of the SS18-SSX transcriptional complex [20]. Concordantly with our present data, *SS18-SSX* knock-out has been reported to upregulate genes in the “regulation of wound healing”, “positive regulation of angiogenesis” and “positive regulation of epithelial cell migration” enriched categories [90]. Actually, activation of pathways promoting tumor progression might also be related to the negative outcome of SS18-SSX breakpoint peptide vaccines in SS patients [124].

SS, which is also considered a stem cell malignancy resulting from dysregulation of self-renewal and multi-lineage differentiation capacities induced by SS18-SSX oncoproteins, displays expression of neural tissue-related genes [1, 44, 53, 125]. Noteworthy, ERK pathway and EGR1 are known to play a relevant role in neuronal survival and plasticity [109] and heparanase has been implicated in brain development and neural cellular differentiation [126]. Our observations in SS models are reminiscent of findings in the AML context showing that the chimeric proteins PLZF/RARa and AML-Eto mediated the reduction or loss of heparanase activity, likely as a consequence of impaired myeloid differentiation, and that treatment with the HDACi trichostatin A reversed the downregulation of heparanase expression induced by the AML-Eto [127].

The p53 oncosuppressor, which also plays a relevant role in processes of neural differentiation [128], is an additional player in the complex network regulating SS cell response to HDACi. P53 acetylation induced by the HDACi trichostatin A has been found to prevent the death of mouse primary cortical neurons [129]. Indeed, by altering protein conformation, stability and interactive properties with gene promoters and proteins, acetylation of p53 governs its transcriptional activity and selection of growth inhibitory versus apoptotic gene targets [11, 85, 130]. ERK, EGR1 and p53-mediated pathways, through their multiple levels of interconnection, play a central role in the balance determining HDACi-induced cell death [67, 130, 131]. Moreover, p53, in cooperation with HDAC, is recognized as a negative regulator of heparanase expression [84]. Histone acetylation at the *TP53* promoter has been proposed as mechanism underlying abrogation of p53-mediated transcriptional repression of heparanase induced by trichostatin A [84]. Our present data, showing that, similarly to the MDM2 inhibitor nutlin-3, the HDACi induced p53 acetylation/stabilization associated with EGR1 and heparanase upregulation, provided an additional mechanism likely contributing to HDACi-induced upregulation of the β-endoglycosidase in SS cells harboring wt *TP53*. Such interpretation is supported by a previous report describing p53 accumulation associated with EGR1 and heparanase upregulation [86]. Moreover, the involvement of p53 acetylation in *HPSE* upregulation induced by HDACi was further sustained by our present observations in *EGR1* silenced cells. Heparanase induction in SS cells as a consequence of HDACi-mediated acetylation/stabilization of wt p53 is of potential translational relevance taking into consideration that SS commonly harbor wt *TP53* [102, 103, 132]*.*

Besides showing that heparanase expression could be epigenetically regulated in SS cells, our data evidenced that the endo-β-D glycosidase, present also in the nucleus of SS cells, may act in turn as an epigenetic regulator promoting histone acetylation, an effect hampered by molecular and pharmacological targeting of the enzyme. The heparanase/HSPG system has been implicated in gene expression regulation by modulation of HAT/HDAC and methylase/demethylase activities as well as by direct interference with the transcriptional machinery [79, 94, 96, 97]*.* Although nuclear expression of heparanase has been associated with differentiation in some tumors [79], the functional role of the endoglycosidase in the nucleus is far from being fully elucidated. Our findings are in line with reports showing the involvement of heparanase in chromatin remodeling through histone posttranslational modifications in different cellular contexts [35, 96, 97, 133–136]. Mechanistically, it has been proposed that, through its degrading activity of nuclear HS, heparanase relieves HS-mediated inhibition of HAT [34]. Our findings using the small molecule OGT2115 and the heparin derivative SST0001 confirmed in SS cells previous observations [33–35] that pharmacological heparanase inhibition could impact histone acetylation as well as heparanase nuclear localization. As concerns heparin derivatives, also acting as HS mimetics, a direct inhibition of the p300 HAT activity in the nucleus has been demonstrated for the 2-O,3-O desulfated heparin CX-01 (ODSH, [136]), whereas the effect of SST0001 is thought to be indirect and ascribed to heparanase inhibition [34]. Since the ability of most heparanase inhibitors to enter the nucleus has not been definitely clarified, it is plausible that drug-induced loss of nuclear heparanase is in some cases the consequence of binding and blocking the enzyme in the cytoplasm [35]. Our present findings, showing a clear nuclear localization of a biotinylated SST0001 derivative, suggest the potential of SST0001-like HS mimetics to directly affect nuclear heparanase and HS functions. Our data suggest that these drug effects could be mediated, at least in part, by modulation of localization/activity of different forms of heparanase present at the nuclear level. However, the role of single nuclear heparanase entities remains to be elucidated.

HS mimetics are an emerging class of antitumor drugs characterized by a complex mechanism of action based on interference with both heparanase and HSPGs’ functions, thereby potentially affecting tumor deregulated and microenvironment-dependent pathways [83]. Our previous studies showed the ability of compounds of this class to potentiate the antitumor efficacy of targeted and cytotoxic drugs in sarcoma models including SS [37, 59, 60]. Here, we provided evidence of a positive cooperation between HDACi and the HS mimetic/heparanase inhibitor SST0001 in both in vitro and in vivo tumor models. HS mimetics may contribute to improve SS responsiveness to HDACi by acting at multiple levels. We demonstrated that heparanase inhibition intercepted the ERK/EGR1/heparanase cytoprotective loop induced by HDACi cell treatment in SS cells. These findings are in accordance with the inhibition of ERK-EGR1-mediated *HPSE* induction observed in HCC cells exposed to the heparanase inhibitor PI-88 [75]. In addition to heparanase, HS mimetics were found to target the cell signaling, supported by HSPGs, of receptor tyrosine kinases relevant in angiogenesis and in the pathobiology of various sarcoma subtypes [28, 29, 137]. As both EGR1 and the β-D-endoglycosidase play crucial roles in angiogenesis [30, 32, 68, 83], we cannot exclude a contribution of angiogenesis inhibition by SST0001 on the growth delay of orthotopic CME-1 xenografts in mice. In addition, the combined treatment enhanced the inhibition of AKT, a key survival effector in SS cells [1]. This effect, together with the abrogation of the HDACi induced ERK activation, likely contributed to the proapoptotic effect in treated cells.

These findings demonstrated the feasibility of this rationale-based approach. Combinations of next-generation HDACi and HS mimetics/heparanase inhibitors endowed with improved pharmacological profiles are worthy of further investigation.

## Conclusions

**Fig. 9:**
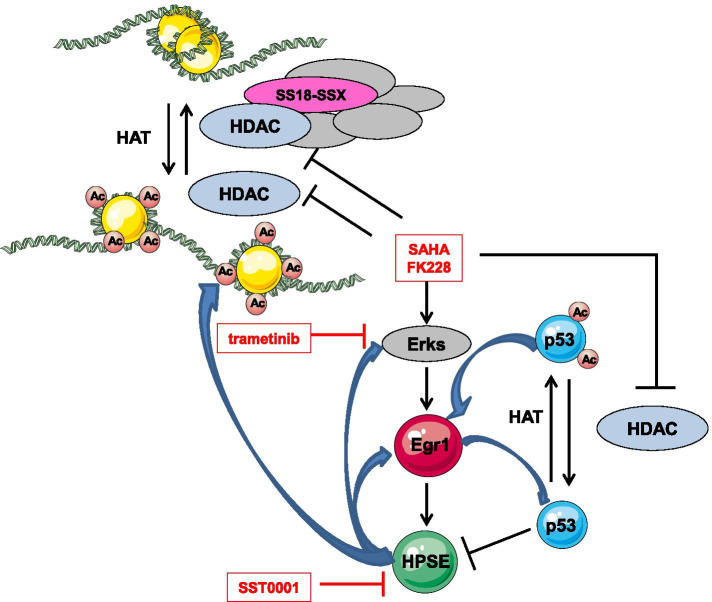
Schematic representation of the proposed HDACi activated auto-sustaining pro-survival loop and its blockade by co-treatment with ERK pathway and heparanase inhibitors in SS cells. This figure was prepared using tools from Servier Medical Art (http://www.servier.fr/servier-medical-art)

This study showed the activation of the ERK-EGR1-heparanase cytoprotective loop induced by HDACi in SS cells through gene expression modulation enhanced by histone and p53 acetylation (Fig. 9). Counteracting this pathway activation by combining a MEK inhibitor or a heparanase inhibitor with HDACi increased the antiproliferative effect enhancing cell death. The positive interaction between SST0001 and SAHA was reflected in vivo by a significant delay of tumor growth in an orthotopic SS model. Overall, our data suggest that the two drugs cooperate, at least partially, at the nuclear level where the heparanase inhibitor was shown to downmodulate heparanase affecting its localization and histone acetylation. These findings provide a rational base to potentially improve the efficacy of HDACi therapies in SS by applying combinatory strategies based on the use of ERK pathway and heparanase inhibitors. The transferability potential of the proposed approaches is indicated by the involvement of classes of agents already clinically available or under clinical evaluation.

## Supplementary Information

**Additional file 1.**

## Data Availability

Not applicable.

## Abbreviations

ER: Endoplasmic reticulum
HAT: Histone acetyltransferase
HDAC: Histone deacetylase
HDACi: Histone deacetylase inhibitor
HGNC: Human Genome Organization (HUGO) Gene Nomenclature Committee
HS: Heparan sulfate
HSPG: Heparan sulfate proteoglycans
PRC: Polycomb repressive complex
SS: Synovial sarcoma
SWI/SNF: Switch/Sucrose non-fermentable complex
