# Supplementary material for: Upregulation of ERK-EGR1-heparanase axis by HDAC inhibitors provides targets for rational therapeutic intervention in synovial sarcoma
Source: J Exp Clin Cancer Res. 2021 Dec 2;40:381. doi: 10.1186/s13046-021-02150-y (PMC8638516; doi:10.1186/s13046-021-02150-y)
Supplement: Supplementary file 1 — Additional file 1. [file 13046_2021_2150_MOESM1_ESM.pptx]

## Slide 1
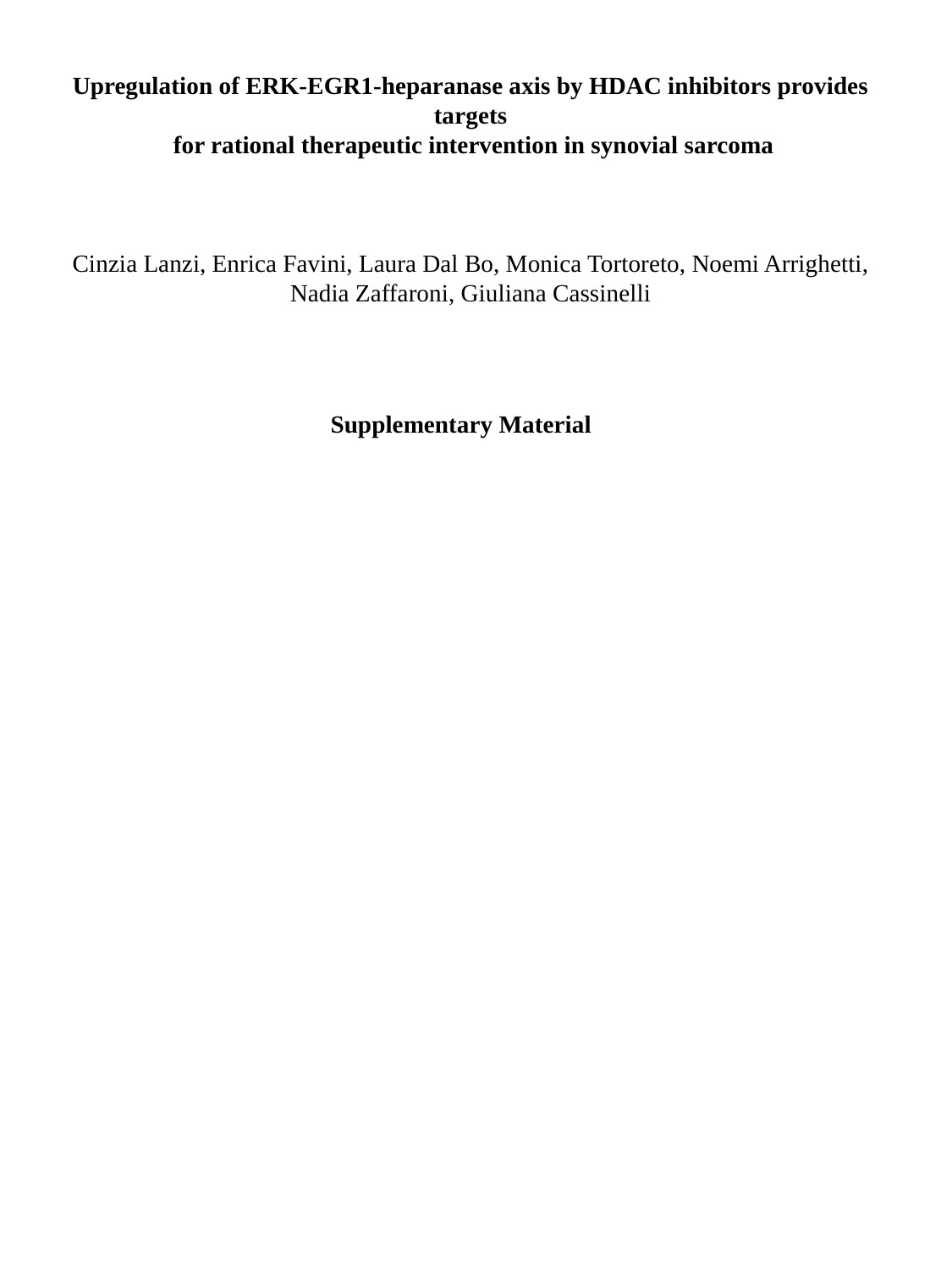

# Upregulation of ERK-EGR1-heparanase axis by HDAC inhibitors provides targets for rational therapeutic intervention in synovial sarcoma   Cinzia Lanzi, Enrica Favini, Laura Dal Bo, Monica Tortoreto, Noemi Arrighetti, Nadia Zaffaroni, Giuliana Cassinelli
Supplementary Material

## Slide 2
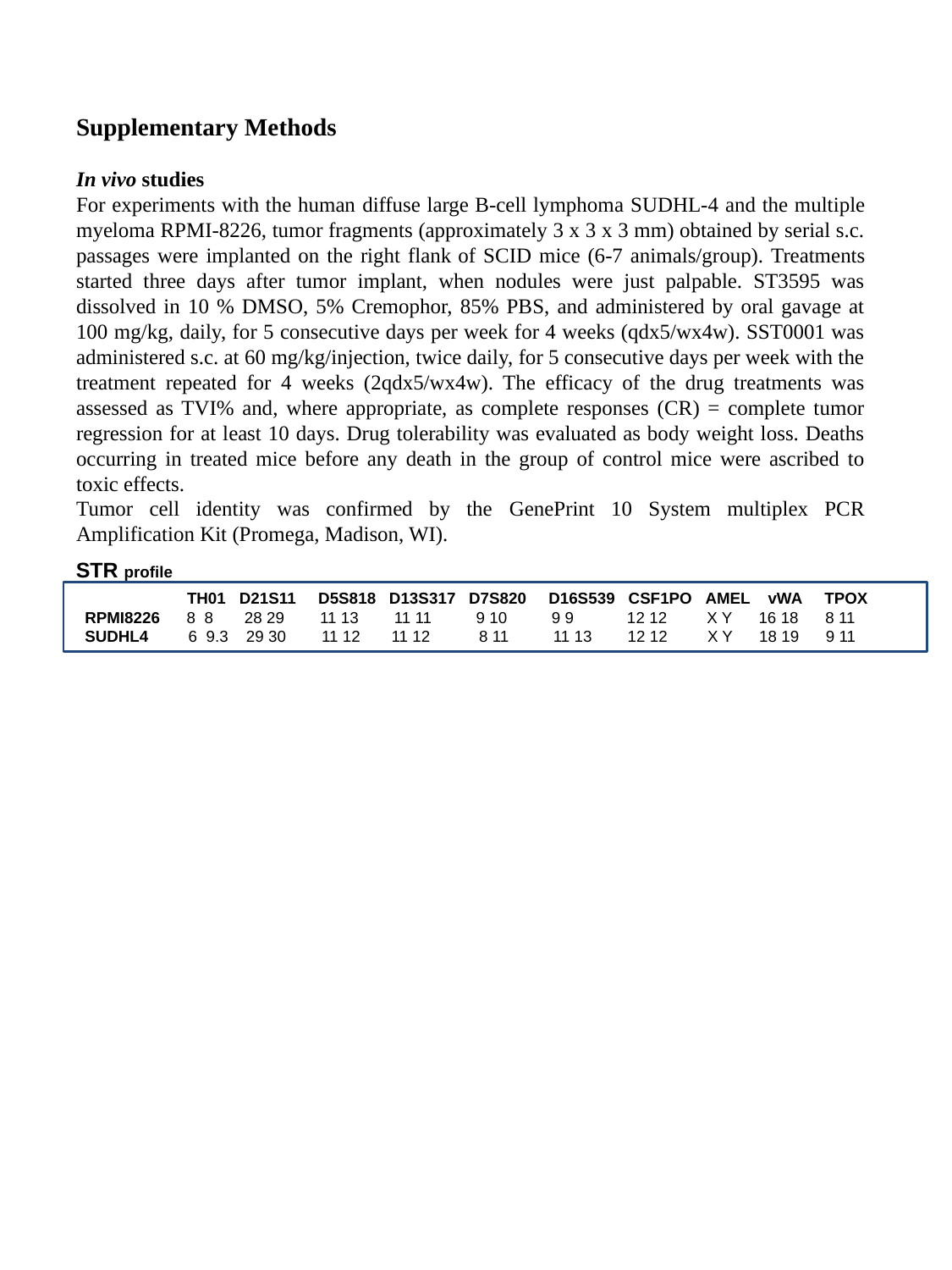

Supplementary Methods
In vivo studies
For experiments with the human diffuse large B-cell lymphoma SUDHL-4 and the multiple myeloma RPMI-8226, tumor fragments (approximately 3 x 3 x 3 mm) obtained by serial s.c. passages were implanted on the right flank of SCID mice (6-7 animals/group). Treatments started three days after tumor implant, when nodules were just palpable. ST3595 was dissolved in 10 % DMSO, 5% Cremophor, 85% PBS, and administered by oral gavage at 100 mg/kg, daily, for 5 consecutive days per week for 4 weeks (qdx5/wx4w). SST0001 was administered s.c. at 60 mg/kg/injection, twice daily, for 5 consecutive days per week with the treatment repeated for 4 weeks (2qdx5/wx4w). The efficacy of the drug treatments was assessed as TVI% and, where appropriate, as complete responses (CR) = complete tumor regression for at least 10 days. Drug tolerability was evaluated as body weight loss. Deaths occurring in treated mice before any death in the group of control mice were ascribed to toxic effects.
Tumor cell identity was confirmed by the GenePrint 10 System multiplex PCR Amplification Kit (Promega, Madison, WI).
STR profile
  TH01   D21S11     D5S818   D13S317   D7S820     D16S539   CSF1PO   AMEL   vWA     TPOX
 RPMI8226 8 8 28 29   11 13 11 11 9 10   9 9 12 12 X Y 16 18   8 11
 SUDHL4 6 9.3 29 30   11 12 11 12 8 11   11 13 12 12 X Y 18 19   9 11

## Slide 3
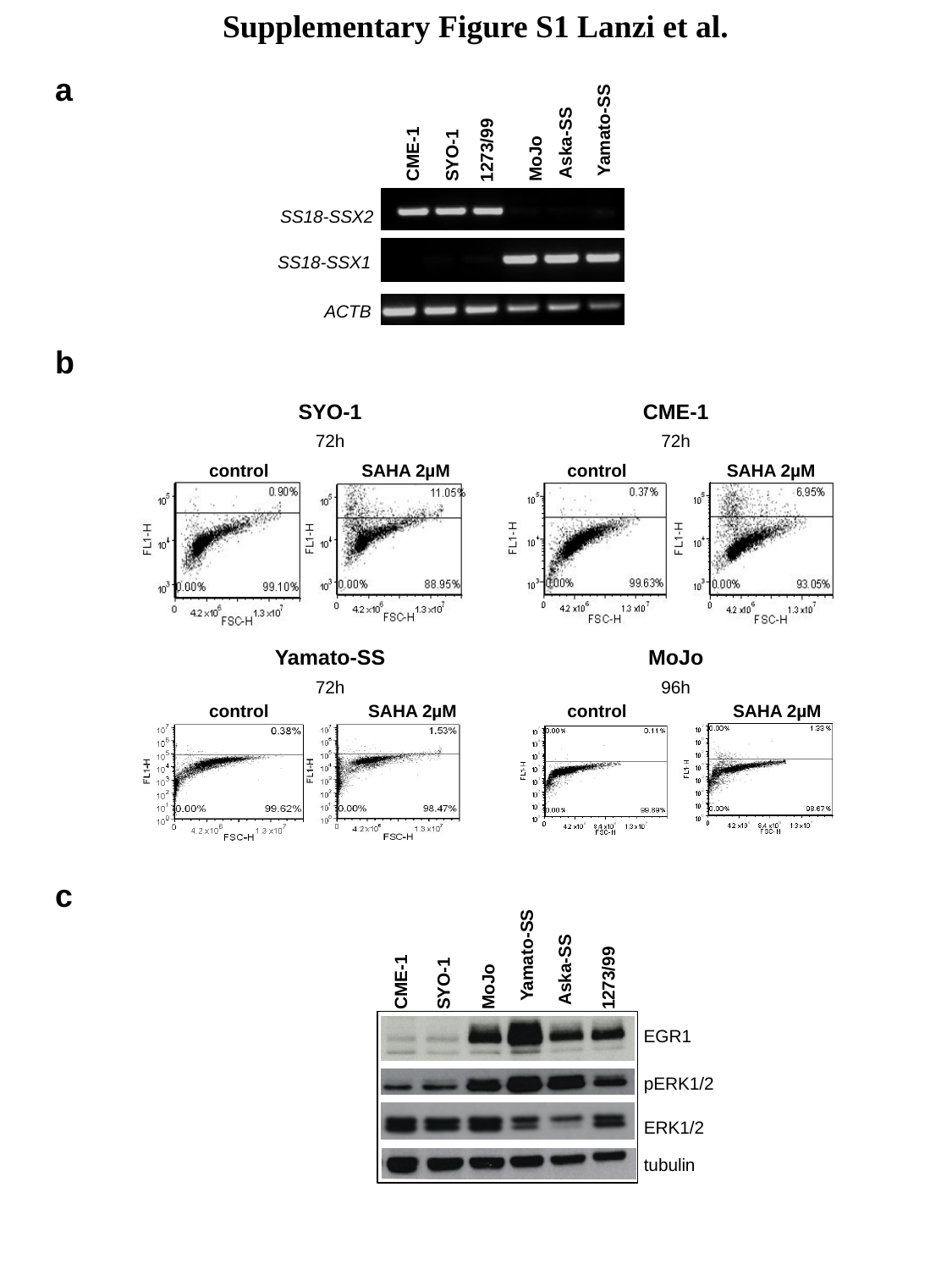

Supplementary Figure S1 Lanzi et al.
a
Yamato-SS
Aska-SS
1273/99
CME-1
SYO-1
MoJo
SS18-SSX2
SS18-SSX1
ACTB
b
SYO-1
CME-1
72h
72h
control
SAHA 2µM
control
SAHA 2µM
Yamato-SS
MoJo
72h
96h
control
SAHA 2µM
control
SAHA 2µM
c
Yamato-SS
Aska-SS
1273/99
CME-1
SYO-1
MoJo
EGR1
pERK1/2
ERK1/2
tubulin

## Slide 4
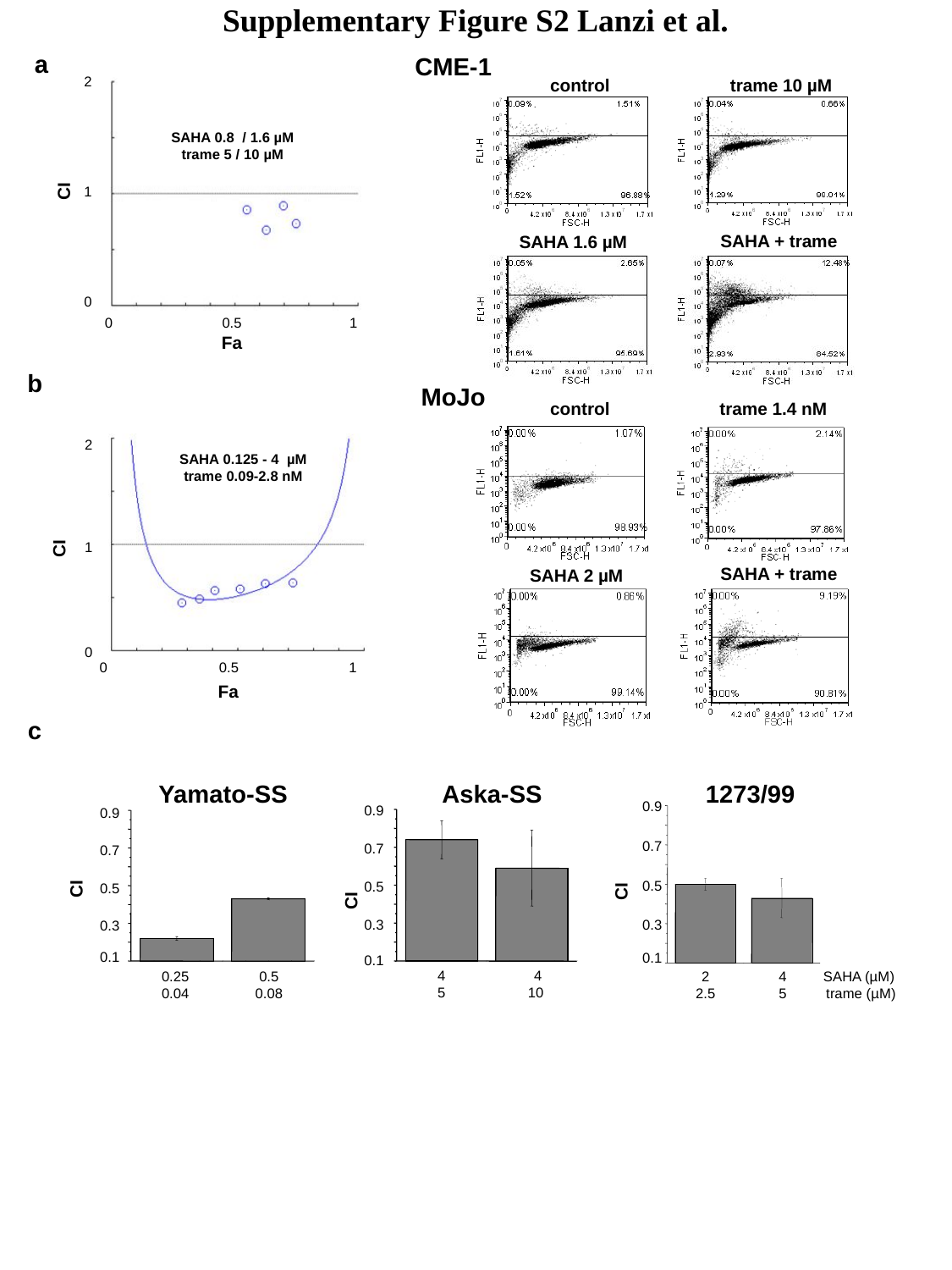

Supplementary Figure S2 Lanzi et al.
a
CME-1
2
CI
1
0
0
0.5
1
Fa
control
trame 10 µM
SAHA 0.8 / 1.6 µM
trame 5 / 10 µM
SAHA + trame
SAHA 1.6 µM
b
MoJo
control
trame 1.4 nM
2
CI
1
0
0
0.5
1
Fa
SAHA 0.125 - 4 µM
trame 0.09-2.8 nM
SAHA + trame
SAHA 2 µM
c
Yamato-SS
Aska-SS
1273/99
0.9
0.7
0.5
0.3
0.1
0.25
0.04
 0.5
 0.08
0.9
0.7
0.5
0.3
0.1
0.9
0.7
0.5
0.3
0.1
CI
4
5
 4
10
CI
CI
SAHA (µM)
trame (µM)
2
2.5
4
5

## Slide 5
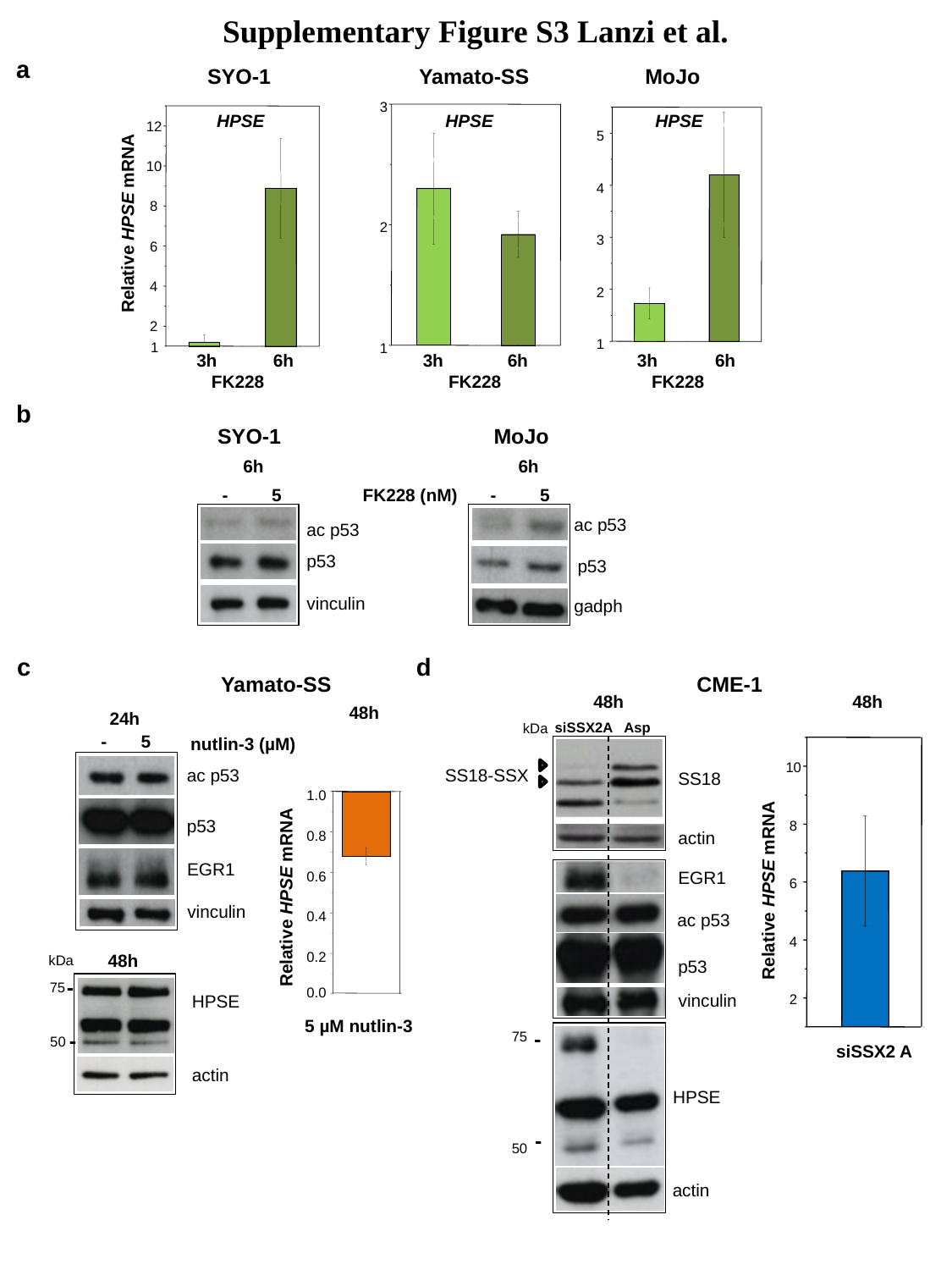

Supplementary Figure S3 Lanzi et al.
a
SYO-1
Yamato-SS
MoJo
3
2
1
12
10
8
6
4
2
1
5
4
3
2
1
HPSE
HPSE
HPSE
Relative HPSE mRNA
3h
6h
3h
6h
3h
6h
FK228
FK228
FK228
b
SYO-1
MoJo
6h
6h
- 5
FK228 (nM)
- 5
ac p53
ac p53
p53
p53
vinculin
gadph
c
d
Yamato-SS
CME-1
48h
48h
24h
48h
siSSX2A Asp
 kDa
SS18-SSX
SS18
actin
EGR1
ac p53
p53
vinculin
-
75
HPSE
-
50
actin
- 5
nutlin-3 (µM)
10
8
6
Relative HPSE mRNA
4
2
siSSX2 A
ac p53
1.0
0.8
0.6
0.4
0.2
0.0
p53
EGR1
Relative HPSE mRNA
vinculin
48h
 kDa
-
75
HPSE
5 µM nutlin-3
-
50
actin

## Slide 6
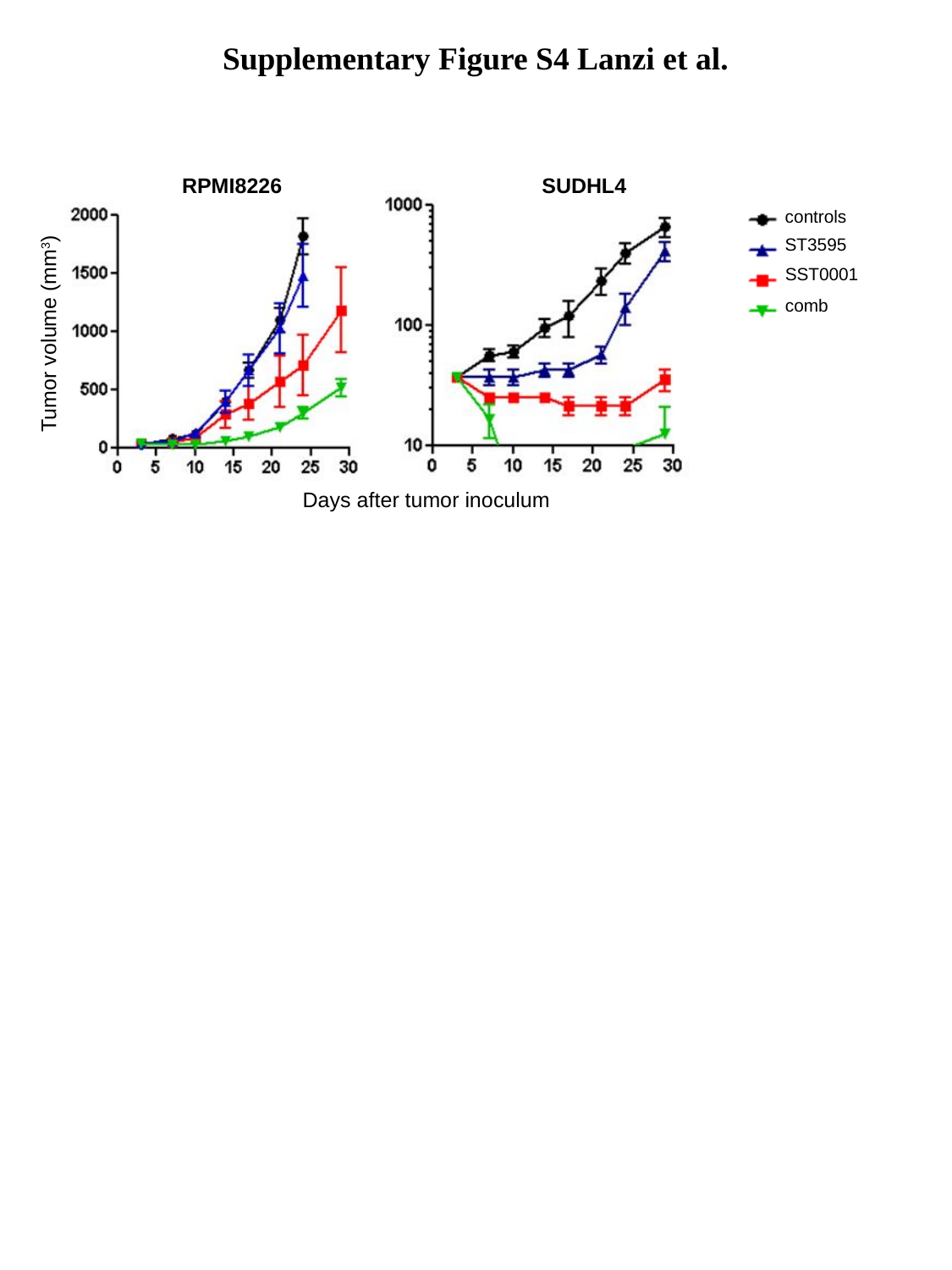

Supplementary Figure S4 Lanzi et al.
RPMI8226
SUDHL4
controls
ST3595
SST0001
comb
Tumor volume (mm3)
Days after tumor inoculum

## Slide 7
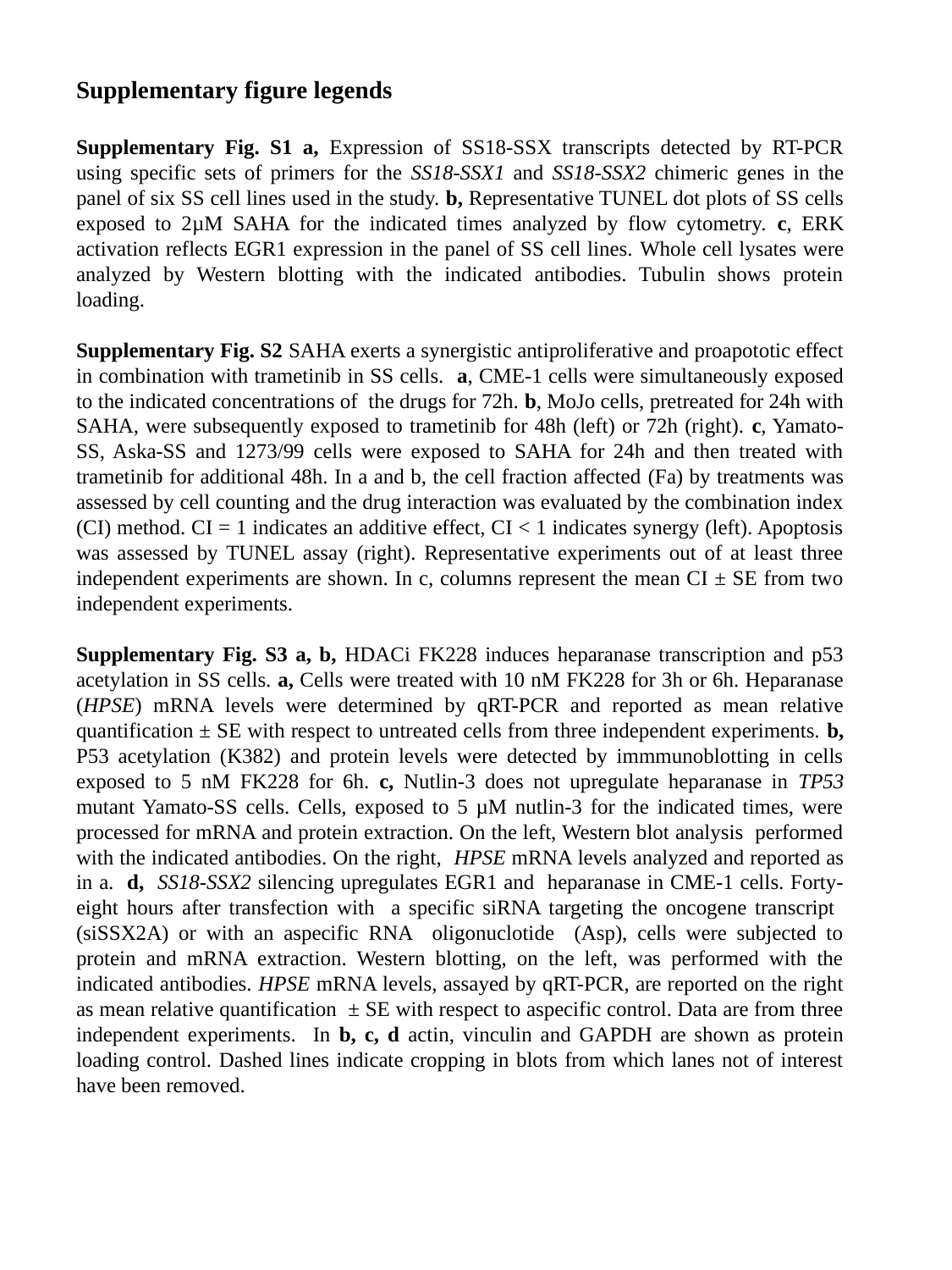

Supplementary figure legends
Supplementary Fig. S1 a, Expression of SS18-SSX transcripts detected by RT-PCR using specific sets of primers for the SS18-SSX1 and SS18-SSX2 chimeric genes in the panel of six SS cell lines used in the study. b, Representative TUNEL dot plots of SS cells exposed to 2µM SAHA for the indicated times analyzed by flow cytometry. c, ERK activation reflects EGR1 expression in the panel of SS cell lines. Whole cell lysates were analyzed by Western blotting with the indicated antibodies. Tubulin shows protein loading.
Supplementary Fig. S2 SAHA exerts a synergistic antiproliferative and proapototic effect in combination with trametinib in SS cells. a, CME-1 cells were simultaneously exposed to the indicated concentrations of the drugs for 72h. b, MoJo cells, pretreated for 24h with SAHA, were subsequently exposed to trametinib for 48h (left) or 72h (right). c, Yamato-SS, Aska-SS and 1273/99 cells were exposed to SAHA for 24h and then treated with trametinib for additional 48h. In a and b, the cell fraction affected (Fa) by treatments was assessed by cell counting and the drug interaction was evaluated by the combination index (CI) method. CI = 1 indicates an additive effect, CI < 1 indicates synergy (left). Apoptosis was assessed by TUNEL assay (right). Representative experiments out of at least three independent experiments are shown. In c, columns represent the mean CI ± SE from two independent experiments.
Supplementary Fig. S3 a, b, HDACi FK228 induces heparanase transcription and p53 acetylation in SS cells. a, Cells were treated with 10 nM FK228 for 3h or 6h. Heparanase (HPSE) mRNA levels were determined by qRT-PCR and reported as mean relative quantification ± SE with respect to untreated cells from three independent experiments. b, P53 acetylation (K382) and protein levels were detected by immmunoblotting in cells exposed to 5 nM FK228 for 6h. c, Nutlin-3 does not upregulate heparanase in TP53 mutant Yamato-SS cells. Cells, exposed to 5 µM nutlin-3 for the indicated times, were processed for mRNA and protein extraction. On the left, Western blot analysis performed with the indicated antibodies. On the right, HPSE mRNA levels analyzed and reported as in a. d, SS18-SSX2 silencing upregulates EGR1 and heparanase in CME-1 cells. Forty-eight hours after transfection with a specific siRNA targeting the oncogene transcript (siSSX2A) or with an aspecific RNA oligonuclotide (Asp), cells were subjected to protein and mRNA extraction. Western blotting, on the left, was performed with the indicated antibodies. HPSE mRNA levels, assayed by qRT-PCR, are reported on the right as mean relative quantification ± SE with respect to aspecific control. Data are from three independent experiments. In b, c, d actin, vinculin and GAPDH are shown as protein loading control. Dashed lines indicate cropping in blots from which lanes not of interest have been removed.

## Slide 8
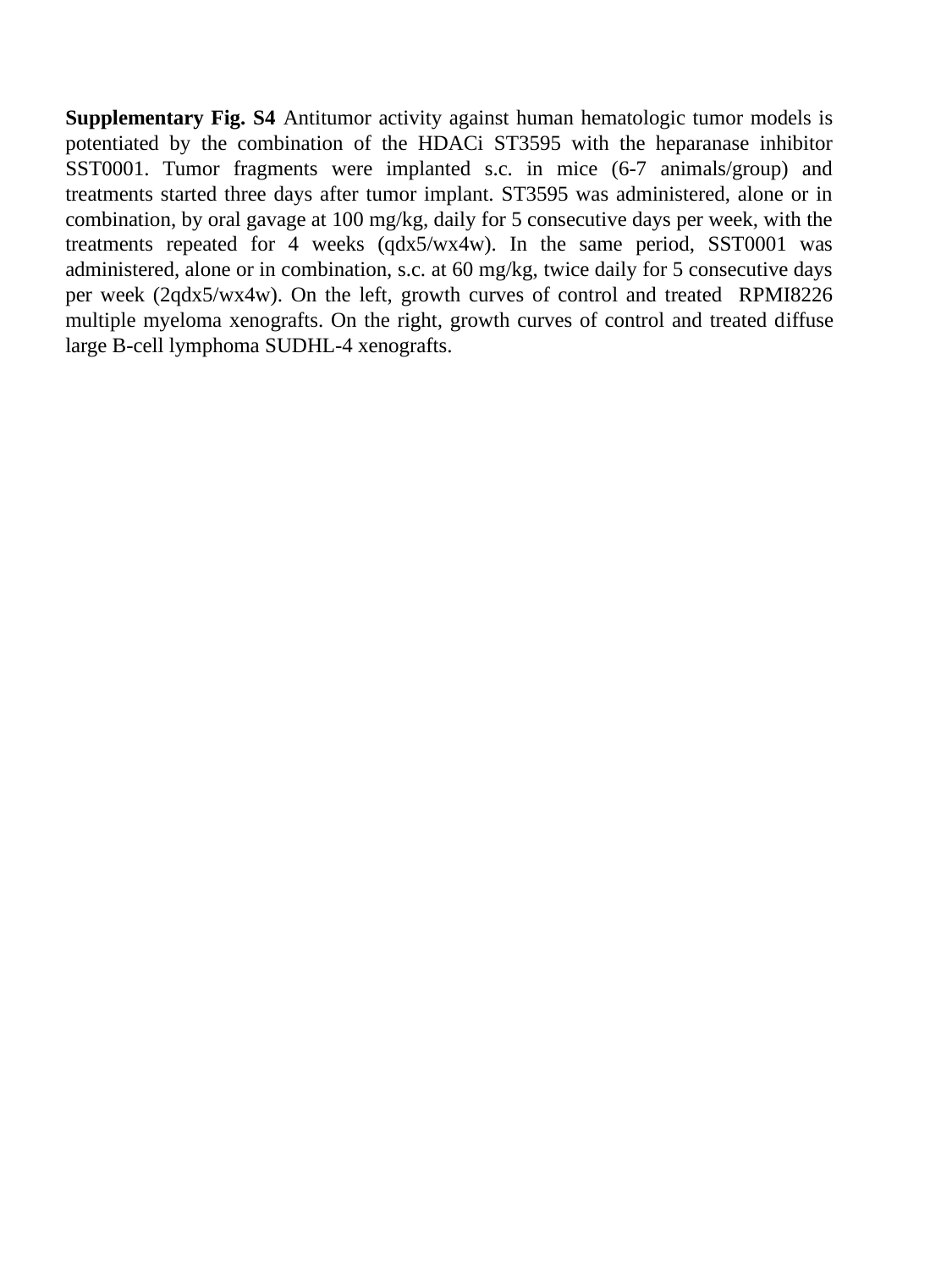

Supplementary Fig. S4 Antitumor activity against human hematologic tumor models is potentiated by the combination of the HDACi ST3595 with the heparanase inhibitor SST0001. Tumor fragments were implanted s.c. in mice (6-7 animals/group) and treatments started three days after tumor implant. ST3595 was administered, alone or in combination, by oral gavage at 100 mg/kg, daily for 5 consecutive days per week, with the treatments repeated for 4 weeks (qdx5/wx4w). In the same period, SST0001 was administered, alone or in combination, s.c. at 60 mg/kg, twice daily for 5 consecutive days per week (2qdx5/wx4w). On the left, growth curves of control and treated RPMI8226 multiple myeloma xenografts. On the right, growth curves of control and treated diffuse large B-cell lymphoma SUDHL-4 xenografts.

## Slide 9
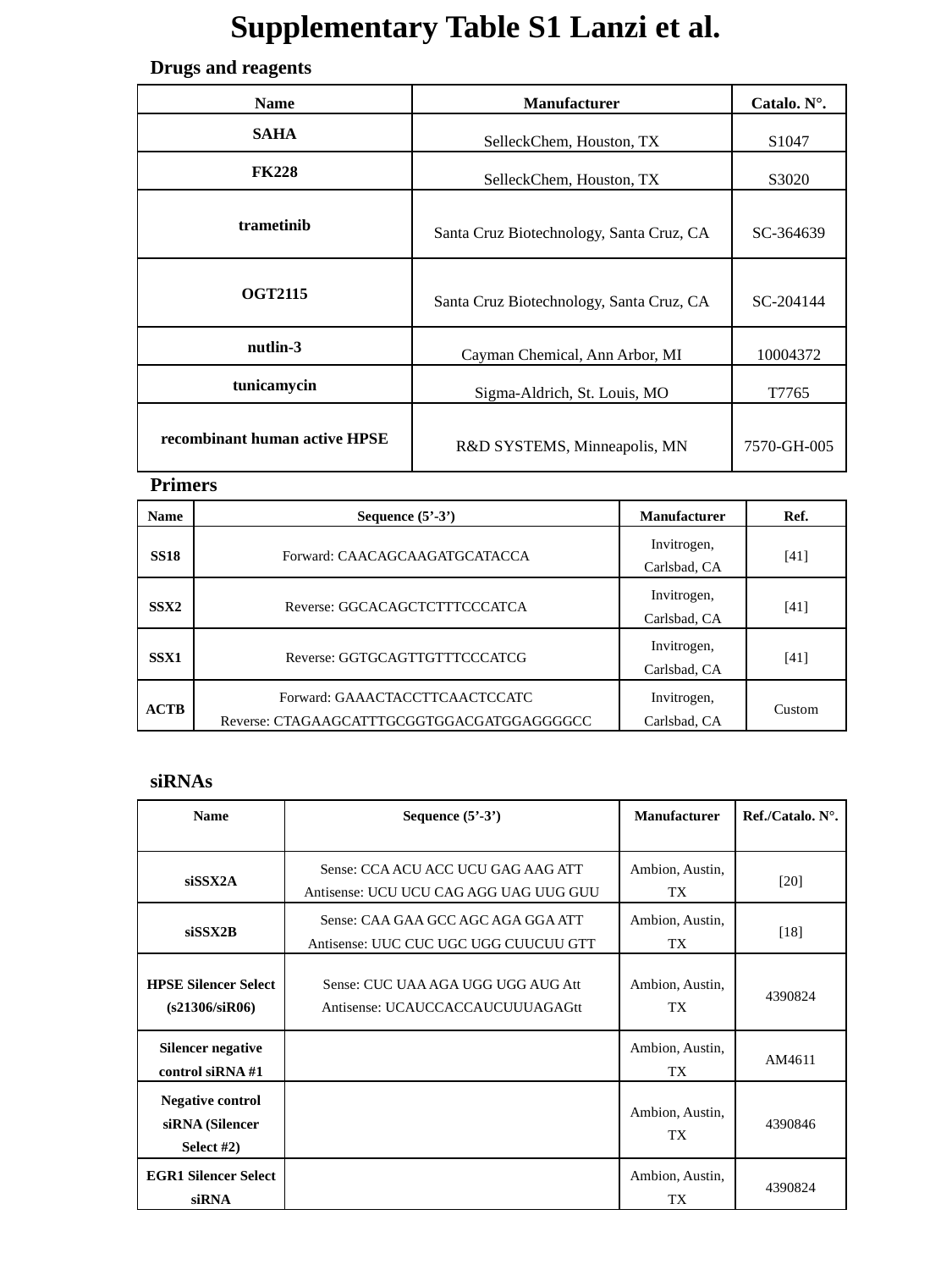

Supplementary Table S1 Lanzi et al.
Drugs and reagents
| Name | Manufacturer | Catalo. N°. |
| --- | --- | --- |
| SAHA | SelleckChem, Houston, TX | S1047 |
| FK228 | SelleckChem, Houston, TX | S3020 |
| trametinib | Santa Cruz Biotechnology, Santa Cruz, CA | SC-364639 |
| OGT2115 | Santa Cruz Biotechnology, Santa Cruz, CA | SC-204144 |
| nutlin-3 | Cayman Chemical, Ann Arbor, MI | 10004372 |
| tunicamycin | Sigma-Aldrich, St. Louis, MO | T7765 |
| recombinant human active HPSE | R&D SYSTEMS, Minneapolis, MN | 7570-GH-005 |
Primers
| Name | Sequence (5’-3’) | Manufacturer | Ref. |
| --- | --- | --- | --- |
| SS18 | Forward: CAACAGCAAGATGCATACCA | Invitrogen, Carlsbad, CA | [41] |
| SSX2 | Reverse: GGCACAGCTCTTTCCCATCA | Invitrogen, Carlsbad, CA | [41] |
| SSX1 | Reverse: GGTGCAGTTGTTTCCCATCG | Invitrogen, Carlsbad, CA | [41] |
| ACTB | Forward: GAAACTACCTTCAACTCCATC Reverse: CTAGAAGCATTTGCGGTGGACGATGGAGGGGCC | Invitrogen, Carlsbad, CA | Custom |
siRNAs
| Name | Sequence (5’-3’) | Manufacturer | Ref./Catalo. N°. |
| --- | --- | --- | --- |
| siSSX2A | Sense: CCA ACU ACC UCU GAG AAG ATT Antisense: UCU UCU CAG AGG UAG UUG GUU | Ambion, Austin, TX | [20] |
| siSSX2B | Sense: CAA GAA GCC AGC AGA GGA ATT Antisense: UUC CUC UGC UGG CUUCUU GTT | Ambion, Austin, TX | [18] |
| HPSE Silencer Select (s21306/siR06) | Sense: CUC UAA AGA UGG UGG AUG Att Antisense: UCAUCCACCAUCUUUAGAGtt | Ambion, Austin, TX | 4390824 |
| Silencer negative control siRNA #1 | | Ambion, Austin, TX | AM4611 |
| Negative control siRNA (Silencer Select #2) | | Ambion, Austin, TX | 4390846 |
| EGR1 Silencer Select siRNA | | Ambion, Austin, TX | 4390824 |

## Slide 10
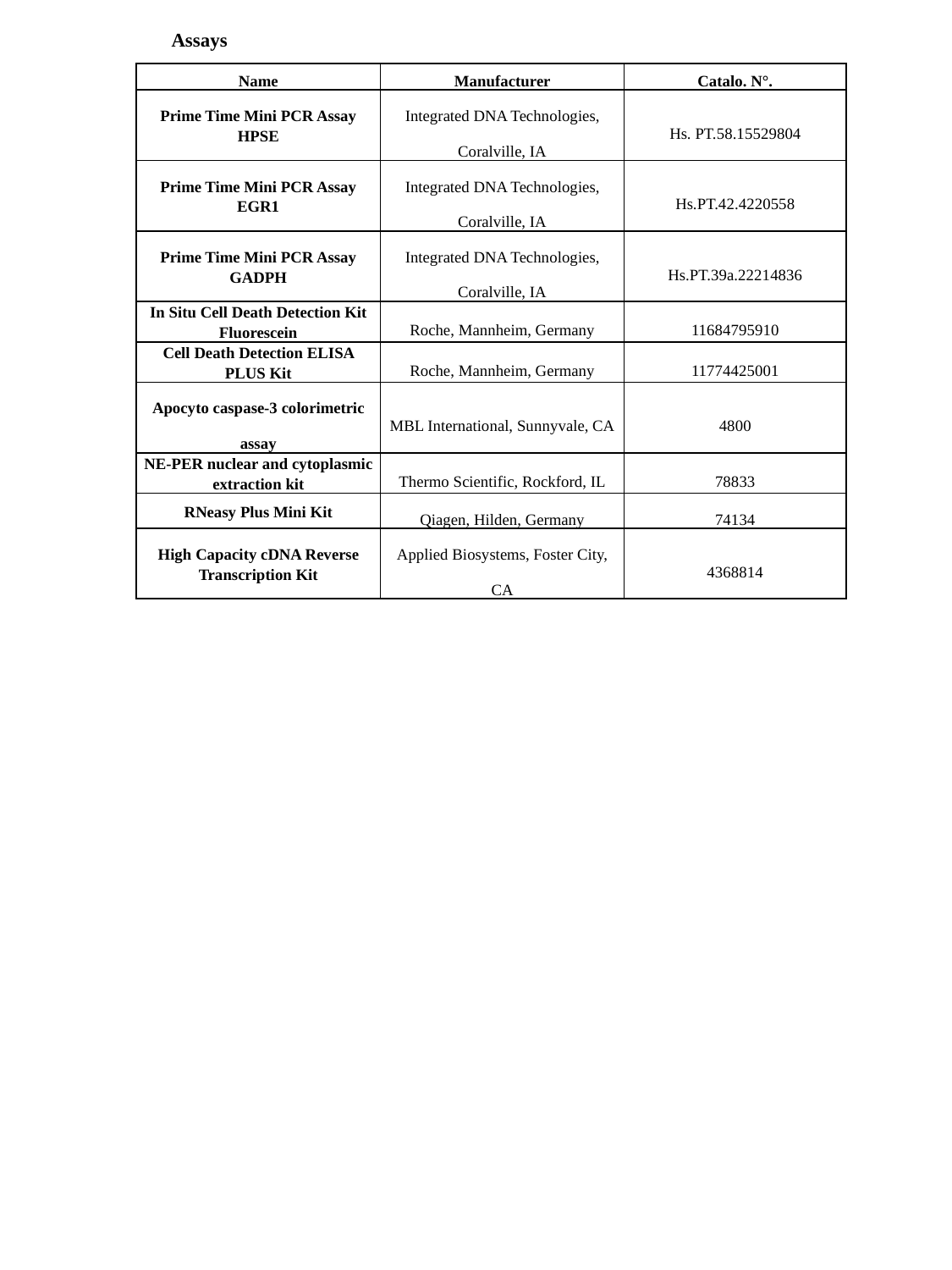

Assays
| Name | Manufacturer | Catalo. N°. |
| --- | --- | --- |
| Prime Time Mini PCR Assay HPSE | Integrated DNA Technologies, Coralville, IA | Hs. PT.58.15529804 |
| Prime Time Mini PCR Assay EGR1 | Integrated DNA Technologies, Coralville, IA | Hs.PT.42.4220558 |
| Prime Time Mini PCR Assay GADPH | Integrated DNA Technologies, Coralville, IA | Hs.PT.39a.22214836 |
| In Situ Cell Death Detection Kit Fluorescein | Roche, Mannheim, Germany | 11684795910 |
| Cell Death Detection ELISA PLUS Kit | Roche, Mannheim, Germany | 11774425001 |
| Apocyto caspase-3 colorimetric assay | MBL International, Sunnyvale, CA | 4800 |
| NE-PER nuclear and cytoplasmic extraction kit | Thermo Scientific, Rockford, IL | 78833 |
| RNeasy Plus Mini Kit | Qiagen, Hilden, Germany | 74134 |
| High Capacity cDNA Reverse Transcription Kit | Applied Biosystems, Foster City, CA | 4368814 |

## Slide 11
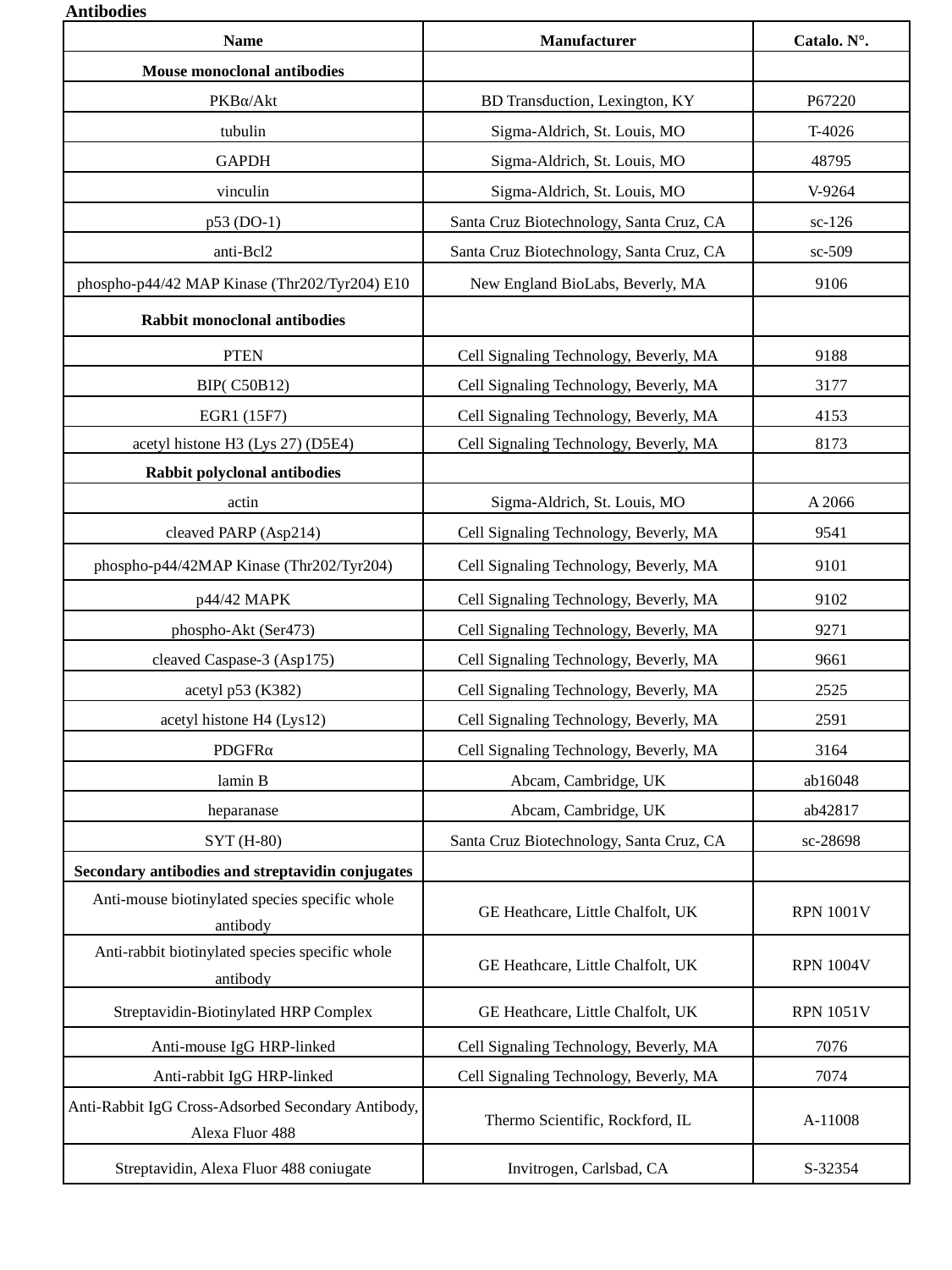

Antibodies
Antibodies
| Name | Manufacturer | Catalo. N°. |
| --- | --- | --- |
| Mouse monoclonal antibodies | | |
| PKBα/Akt | BD Transduction, Lexington, KY | P67220 |
| tubulin | Sigma-Aldrich, St. Louis, MO | T-4026 |
| GAPDH | Sigma-Aldrich, St. Louis, MO | 48795 |
| vinculin | Sigma-Aldrich, St. Louis, MO | V-9264 |
| p53 (DO-1) | Santa Cruz Biotechnology, Santa Cruz, CA | sc-126 |
| anti-Bcl2 | Santa Cruz Biotechnology, Santa Cruz, CA | sc-509 |
| phospho-p44/42 MAP Kinase (Thr202/Tyr204) E10 | New England BioLabs, Beverly, MA | 9106 |
| Rabbit monoclonal antibodies | | |
| PTEN | Cell Signaling Technology, Beverly, MA | 9188 |
| BIP( C50B12) | Cell Signaling Technology, Beverly, MA | 3177 |
| EGR1 (15F7) | Cell Signaling Technology, Beverly, MA | 4153 |
| acetyl histone H3 (Lys 27) (D5E4) | Cell Signaling Technology, Beverly, MA | 8173 |
| Rabbit polyclonal antibodies | | |
| actin | Sigma-Aldrich, St. Louis, MO | A 2066 |
| cleaved PARP (Asp214) | Cell Signaling Technology, Beverly, MA | 9541 |
| phospho-p44/42MAP Kinase (Thr202/Tyr204) | Cell Signaling Technology, Beverly, MA | 9101 |
| p44/42 MAPK | Cell Signaling Technology, Beverly, MA | 9102 |
| phospho-Akt (Ser473) | Cell Signaling Technology, Beverly, MA | 9271 |
| cleaved Caspase-3 (Asp175) | Cell Signaling Technology, Beverly, MA | 9661 |
| acetyl p53 (K382) | Cell Signaling Technology, Beverly, MA | 2525 |
| acetyl histone H4 (Lys12) | Cell Signaling Technology, Beverly, MA | 2591 |
| PDGFRα | Cell Signaling Technology, Beverly, MA | 3164 |
| lamin B | Abcam, Cambridge, UK | ab16048 |
| heparanase | Abcam, Cambridge, UK | ab42817 |
| SYT (H-80) | Santa Cruz Biotechnology, Santa Cruz, CA | sc-28698 |
| Secondary antibodies and streptavidin conjugates | | |
| Anti-mouse biotinylated species specific whole antibody | GE Heathcare, Little Chalfolt, UK | RPN 1001V |
| Anti-rabbit biotinylated species specific whole antibody | GE Heathcare, Little Chalfolt, UK | RPN 1004V |
| Streptavidin-Biotinylated HRP Complex | GE Heathcare, Little Chalfolt, UK | RPN 1051V |
| Anti-mouse IgG HRP-linked | Cell Signaling Technology, Beverly, MA | 7076 |
| Anti-rabbit IgG HRP-linked | Cell Signaling Technology, Beverly, MA | 7074 |
| Anti-Rabbit IgG Cross-Adsorbed Secondary Antibody, Alexa Fluor 488 | Thermo Scientific, Rockford, IL | A-11008 |
| Streptavidin, Alexa Fluor 488 coniugate | Invitrogen, Carlsbad, CA | S-32354 |

## Slide 12
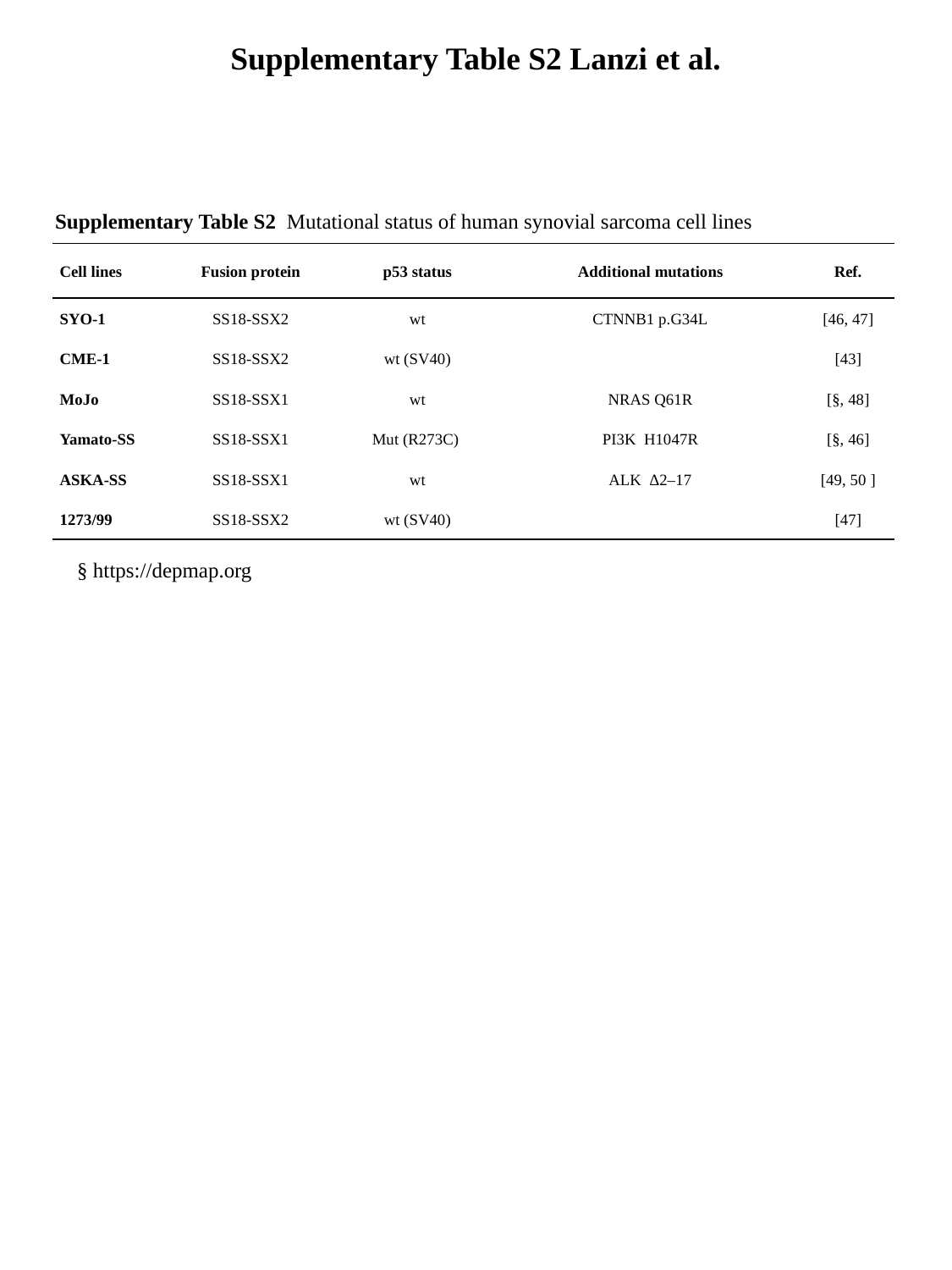

Supplementary Table S2 Lanzi et al.
Supplementary Table S2 Mutational status of human synovial sarcoma cell lines
| Cell lines | Fusion protein | p53 status | Additional mutations | Ref. |
| --- | --- | --- | --- | --- |
| SYO-1 | SS18-SSX2 | wt | CTNNB1 p.G34L | [46, 47] |
| CME-1 | SS18-SSX2 | wt (SV40) | | [43] |
| MoJo | SS18-SSX1 | wt | NRAS Q61R | [§, 48] |
| Yamato-SS | SS18-SSX1 | Mut (R273C) | PI3K H1047R | [§, 46] |
| ASKA-SS | SS18-SSX1 | wt | ALK Δ2–17 | [49, 50 ] |
| 1273/99 | SS18-SSX2 | wt (SV40) | | [47] |
§ https://depmap.org

## Slide 13
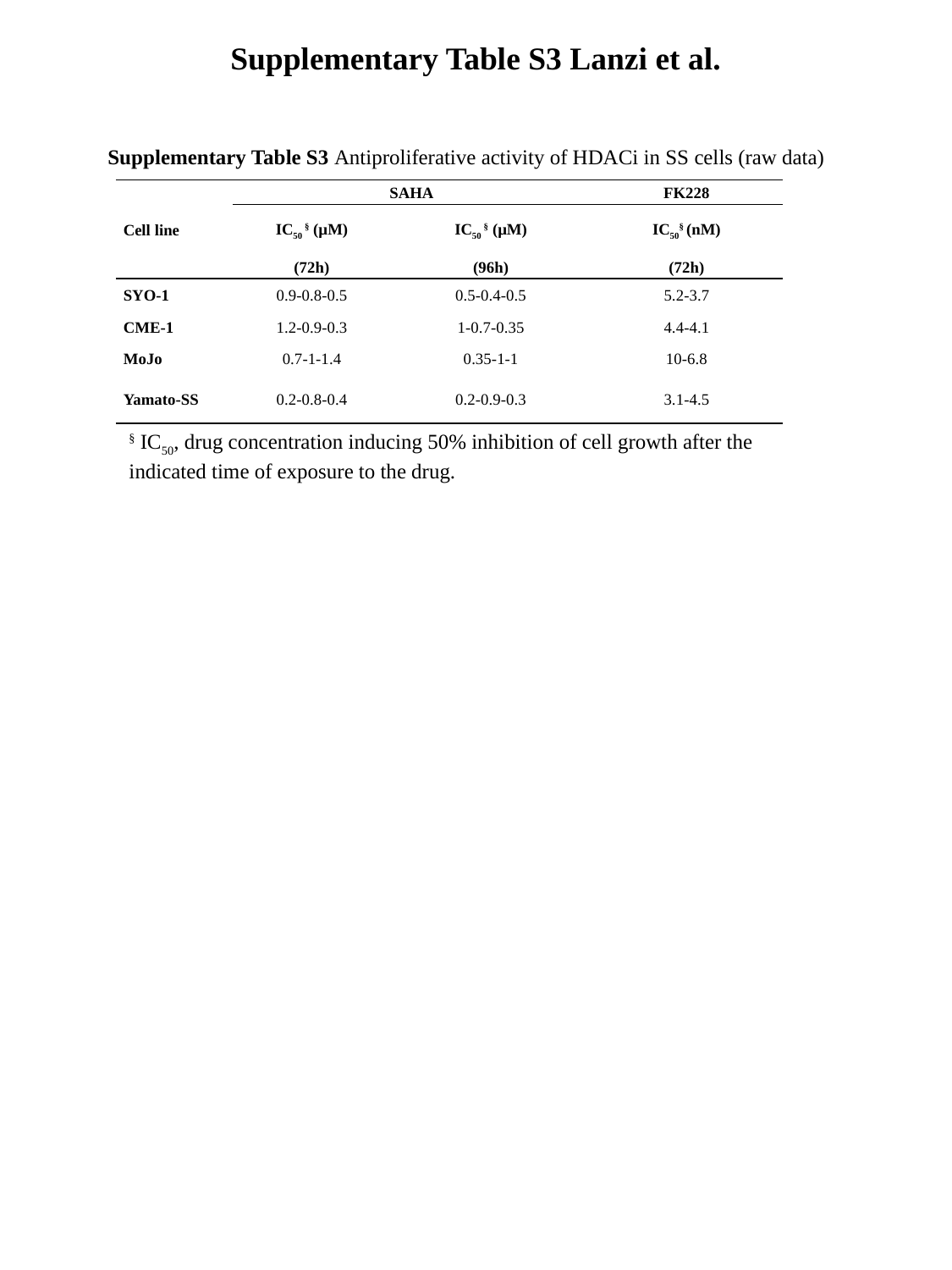

Supplementary Table S3 Lanzi et al.
Supplementary Table S3 Antiproliferative activity of HDACi in SS cells (raw data)
| Cell line | SAHA | | FK228 |
| --- | --- | --- | --- |
| | IC50 § (µM) | IC50 § (µM) | IC50§ (nM) |
| | (72h) | (96h) | (72h) |
| SYO-1 | 0.9-0.8-0.5 | 0.5-0.4-0.5 | 5.2-3.7 |
| CME-1 | 1.2-0.9-0.3 | 1-0.7-0.35 | 4.4-4.1 |
| MoJo | 0.7-1-1.4 | 0.35-1-1 | 10-6.8 |
| Yamato-SS | 0.2-0.8-0.4 | 0.2-0.9-0.3 | 3.1-4.5 |
§ IC50, drug concentration inducing 50% inhibition of cell growth after the indicated time of exposure to the drug.

## Slide 14
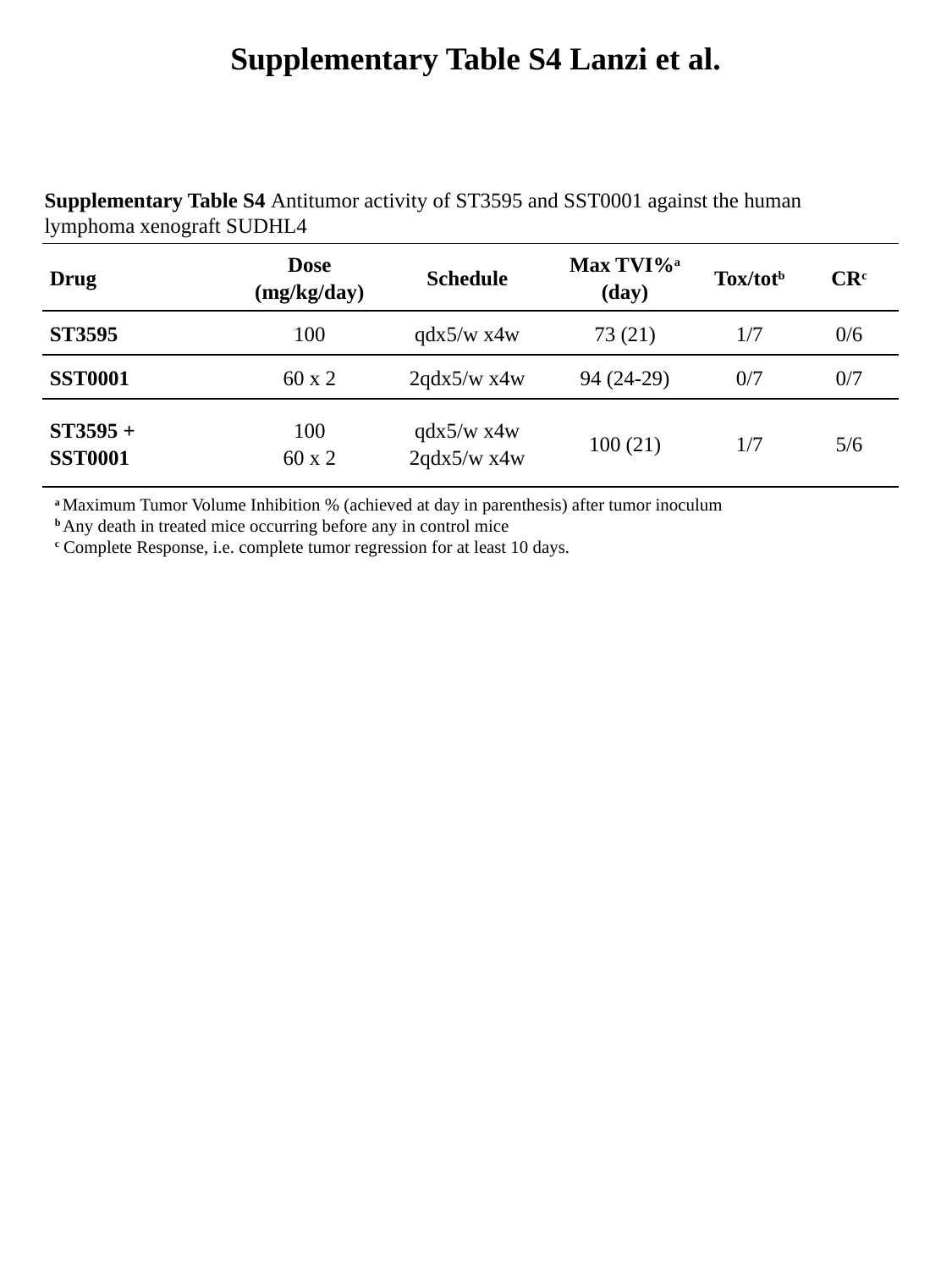

Supplementary Table S4 Lanzi et al.
Supplementary Table S4 Antitumor activity of ST3595 and SST0001 against the human lymphoma xenograft SUDHL4
| Drug | Dose (mg/kg/day) | Schedule | Max TVI%a (day) | Tox/totb | CRc |
| --- | --- | --- | --- | --- | --- |
| ST3595 | 100 | qdx5/w x4w | 73 (21) | 1/7 | 0/6 |
| SST0001 | 60 x 2 | 2qdx5/w x4w | 94 (24-29) | 0/7 | 0/7 |
| ST3595 + SST0001 | 100 60 x 2 | qdx5/w x4w 2qdx5/w x4w | 100 (21) | 1/7 | 5/6 |
a Maximum Tumor Volume Inhibition % (achieved at day in parenthesis) after tumor inoculum
b Any death in treated mice occurring before any in control mice
c Complete Response, i.e. complete tumor regression for at least 10 days.
